# Adverse Event Reporting Patterns of Tobacco Cessation Medications in Korea: A Disproportionality Analysis

**DOI:** 10.3390/healthcare14203479

**Published:** 2026-10-16

**Authors:** Jeongah Min, Eunah Cho, Sooyoung Shin, Yeo Jin Choi

**Affiliations:** 1 Department of Regulatory Science, Graduate School, Kyung Hee University, Seoul 02447, Republic of Korea; 2 Institute of Regulatory Innovation Through Science (IRIS), Kyung Hee University, Seoul 02447, Republic of Korea; 3 Department of Pharmacy, College of Pharmacy and Integrated Pharmaceutical Sciences, Kyung Hee University, Seoul 02447, Republic of Korea; 4 Department of Pharmacy, College of Pharmacy, Ajou University, Suwon 16499, Republic of Korea; 5 Research Institute of Pharmaceutical Science and Technology (RIPST), Ajou University, Suwon 16499, Republic of Korea

**Keywords:** tobacco cessation, pharmacovigilance, adverse drug events, medication safety, pharmacist

## Abstract

**Background/Objectives**: This study aimed to characterize adverse event (AE) reporting patterns associated with tobacco cessation medications using nationwide pharmacovigilance data from Korea. **Methods**: A retrospective pharmacovigilance study on tobacco cessation medications using the Korea Adverse Event Reporting System database (KIDS KAERS DB) was conducted. Descriptive analyses were performed to summarize demographic and reporting characteristics and AE patterns. Disproportionality analyses, including proportional reporting ratios (PRR), reporting odds ratios (ROR), and information components (IC), were performed to detect potential signals of disproportionate reporting. **Results**: A total of 779 individual case safety reports (ICSRs) involving tobacco cessation medications were included. Nicotine replacement therapy (NRT) accounted for 606 ICSRs (77.79%), followed by varenicline (*n* = 150, 19.26%) and bupropion (*n* = 23, 2.95%). Age information was unavailable for 558 ICSRs (71.63%). Among ICSRs with documented age information, the 50–59-year age group was the most frequently represented. Fifty-two serious ICSRs (6.68%) were identified, comprising 49 ICSRs involving NRT and 3 involving bupropion. SOC-level disproportionality analyses identified signals of disproportionate reporting for psychiatric disorders for bupropion and psychiatric and gastrointestinal disorders for varenicline. All identified signals remained detectable after excluding reports involving individuals aged <18 years, whereas causality-based restriction altered the detected signal patterns. **Conclusions**: This nationwide pharmacovigilance study identified medication-specific adverse event reporting patterns across NRT, bupropion, and varenicline. While these findings do not establish causal relationships, the observed differences in reporting patterns provide pharmacovigilance insights into the post-marketing reporting patterns of tobacco cessation pharmacotherapies and support further evaluation using pharmacoepidemiologic studies with appropriate exposure denominators.

## 1. Introduction

Tobacco use remains a major preventable cause of morbidity and mortality worldwide, contributing substantially to the global burden of cardiovascular disease, cancer, and respiratory disorders [1]. More than 8 million deaths are attributed to tobacco use annually, including over 7 million from direct tobacco use [1,2,3]. Despite ongoing tobacco control efforts, many individuals struggle to quit due to nicotine dependence and withdrawal symptoms [4,5]. Pharmacological interventions, including nicotine replacement therapy (NRT), varenicline, and bupropion, are widely recommended to support smoking cessation and improve quit rates [6,7].

Although these medications are generally effective and well tolerated, each has a distinct safety profile that requires consideration in clinical practice. NRT is generally well tolerated, with adverse effects primarily related to the formulation, such as skin irritation with transdermal patches and oral irritation with gum or tablets [8]. Earlier post-marketing reports raised concerns regarding neuropsychiatric and cardiovascular adverse events (AEs), particularly with varenicline and bupropion [9]. However, the EAGLES randomized trial found no significant increase in moderate-to-severe neuropsychiatric AEs with varenicline or bupropion compared with NRT or placebo, and subsequent analyses found no significant differences in major cardiovascular events among these treatments [10,11]. Consistent with these findings, subsequent regulatory assessment by the European Medicines Agency found no evidence supporting an increased risk of neuropsychiatric events with varenicline [12]. Nevertheless, continued pharmacovigilance remains important for identifying rare or unexpected AEs that may not be fully characterized in clinical trials.

Pharmacovigilance databases provide valuable opportunities to identify rare or unexpected AEs that may not be detected in pre-marketing clinical trials [13]. Spontaneous reporting systems are useful for detecting potential safety signals, although reported associations do not establish causality [14,15,16,17]. Previous pharmacovigilance studies have examined smoking-cessation medications using national and international spontaneous-reporting databases [14,15,16,17]. Previous Korean pharmacovigilance research using the Korea Adverse Event Reporting System database (KAERS DB) has primarily focused on signal detection for varenicline [17], while concurrent evaluation of AE reporting patterns across NRT, bupropion, and varenicline within the same national pharmacovigilance database remains limited. Therefore, the primary objective of this study was to characterize AE reporting patterns associated with NRT, bupropion, and varenicline in the KIDS KAERS DB and to identify potential signals of disproportionate reporting. Secondary objectives were to describe demographic characteristics, seriousness, temporal reporting patterns, and reporter characteristics at the individual case safety report (ICSR) level.

## 2. Materials and Methods

### 2.1. Study Design and Data Collection

A disproportionality analysis using individual case safety reports from KIDS KAERS DB was conducted in accordance with the strengthening the reporting of observational studies in epidemiology (STROBE) guidelines and the Reporting of A Disproportionality Analysis for DrUg Safety Signal Detection using Individual Case Safety Reports in Pharmacovigilance (READUS-PV) recommendations (Tables S1 and S2) [18,19]. This study analyzed adverse drug events (ADEs) related to tobacco cessation products, including bupropion, NRT, and varenicline spontaneously reported to the KIDS KAERS DB, constructed by the Korea Institute of Drug Safety & Risk Management (KIDS, Ministry of Food and Drug Safety), between 1 January 2014 and 31 December 2023. Because formulation and route information were not consistently available throughout the study period, smoking-cessation medications were classified at the active-ingredient level, with nicotine-containing products grouped as NRT. The causality of ADEs was assessed according to the World Health Organization-Uppsala Monitoring Centre (WHO-UMC) criteria [20]. To minimize potential indication bias, particularly for bupropion, only reports associated with tobacco cessation-related diagnoses or treatment indications were included. Reports with missing indication information or with indications that could not be clearly classified as tobacco cessation-related were excluded. Eligible cases were restricted to reports containing tobacco-related Korean Standard Classification Disease (KCD) codes, including nicotine dependence (F17), and tobacco use (Z72.0), or tobacco cessation-related efficacy terms, such as smoking cessation therapy, smoking cessation, tobacco use, tobacco withdrawal symptoms, tobacco dependence, or nicotine-related effects. The prespecified exclusion criteria were reports associated with non-tobacco cessation indications, including major depressive disorder, insomnia, or other psychiatric indications for which bupropion may be prescribed, and reports involving masked (MSK-coded) etiologic medications. The KIDS KAERS DB assigns MSK codes to medication products marketed by fewer than two pharmaceutical companies. The following data were extracted from KIDS KAERS DB: (1) patient demographics (sex and age), (2) medical histories and concurrent medications, and (3) ADE characteristics, including etiologic medication, occurrence date, causality assessment, ADE type, and seriousness. ICSRs were identified using anonymized KAERS report number (KAERS_NO), which was used to link records belonging to the same safety report across the merged tables. After merging the relevant KAERS DB tables, rows with identical values across all variables were considered exact duplicates and were removed. When multiple versions of the same ICSR were available, only the most recent version was retained for analysis in accordance with the KIDS KAERS DB data-use guidelines [21]. Descriptive characteristics and seriousness were summarized at the ICSR level. For preferred-term (PT)-level descriptive analyses, each unique ICSR–drug–PT combination was counted once. For System Organ Class (SOC)-level analyses, multiple PTs belonging to the same SOC within a single ICSR were counted only once, whereas an ICSR could contribute to more than one SOC when different reported events belonged to different SOCs. AE terms in the KIDS KAERS DB were provided as MedDRA lowest level terms (LLTs), with World Health Organization-Adverse Reaction Terminology (WHO-ART) codes provided in the database. PT- and SOC-level analyses were based on the WHO-ART-coded variables using WHO-ART version 092. No conversion between MedDRA and WHO-ART was performed, and MedDRA LLT names were retained only to describe the originally reported adverse-event terms. SAEs were defined as any ADEs involving congenital abnormalities or birth defects, life-threatening conditions, death, a persistent or significant disability or incapacity, hospitalization or prolonged existing hospitalizations, and other medically significant events, in accordance with the International Conference on Harmonization (ICH) E2D guidelines. The study protocol was approved by KIDS (Ministry of Food and Drug Safety) (KIDS KAERS DB 2407A0003), and Kyung Hee University Institutional Review Board (IRB) (No. KHSIRB-25-515(EA), approved 31 October 2025).

### 2.2. Statistical Analysis

Descriptive statistics were performed to summarize the demographic and reporting characteristics of the included ICSRs and AE patterns. Age was expressed as the median and interquartile range (IQR) based on the Kolmogorov–Smirnov normality test. Missing demographic information was not imputed. Age- and sex-specific descriptive analyses were conducted using available information, and the proportion of missing demographic information was reported. For PT-level analyses, each unique ICSR–drug–PT combination was counted once. For SOC-level analyses, each unique ICSR–drug–SOC combination was counted once, such that multiple PTs belonging to the same SOC within a single ICSR did not inflate the SOC count. An ICSR could contribute to more than one SOC when different reported events belonged to different SOCs. For each target medication, the comparator comprised ICSRs involving the other two smoking-cessation medications. Accordingly, the disproportionality estimates represent relative reporting within the three smoking-cessation medication groups rather than comparisons with the entire KAERS database.

Disproportionality analyses were performed for medication–SOC and medication–PT combinations reported in at least three unique ICSRs (A ≥ 3) [22,23]. A signal of disproportionate reporting (SDR) was considered present only when all of the following criteria were met: PRR ≥ 2, ROR ≥ 2, Pearson’s chi-square ≥ 4, and IC025 > 0 [24]. PRR compares the proportion of a specific AE category reported for the target medication with that reported for the comparator medications, whereas ROR compares the corresponding reporting odds [19]. IC is a Bayesian measure of disproportionality on the log_2_ scale that compares observed with expected reporting and incorporates shrinkage through the BCPNN framework [19]. When any cell of the 2 × 2 contingency table contained zero, 0.5 was added to all four cells using the Haldane–Anscombe correction for estimation of the PRR and ROR. Pearson’s chi-square and the Bayesian Confidence Propagation Neural Network (BCPNN) were calculated using the original, uncorrected cell counts (Table 1). For BCPNN analysis, prior parameters were set to α1 = β1 = γ11 = 1 and α = β = 2. The posterior mean E (IC) was used as the IC estimate, with approximate 95% credibility limits calculated as IC025 = E(IC) − 2√V(IC) and IC975 = E(IC) + 2√V(IC) [25,26]. The primary disproportionality analysis included all eligible ICSRs regardless of WHO-UMC causality assessment, including reports with missing or unassessed causality information. The distribution of WHO-UMC causality assessment by medication is summarized in Table S3. A sensitivity analysis was performed after excluding ICSRs with documented age < 18 years while retaining those with missing age information. An additional sensitivity analysis was conducted by restricting drug–event relationships to those classified as “certain”, “probable/likely”, or “possible” according to WHO-UMC criteria to assess the influence of causality-based restriction on SDR detection. All other eligibility criteria and analytical procedures were maintained as in the primary analysis. Statistical analyses were conducted using IBM SPSS Statistics, version 26.0 (IBM Corp., Armonk, NY, USA) and R version 4.5.1 (R Foundation for Statistical Computing, Vienna, Austria). IC and its credibility limits were calculated in R using a custom implementation of the BCPNN equations described above.

## 3. Results

### 3.1. Baseline Characteristics

**Figure 1:**
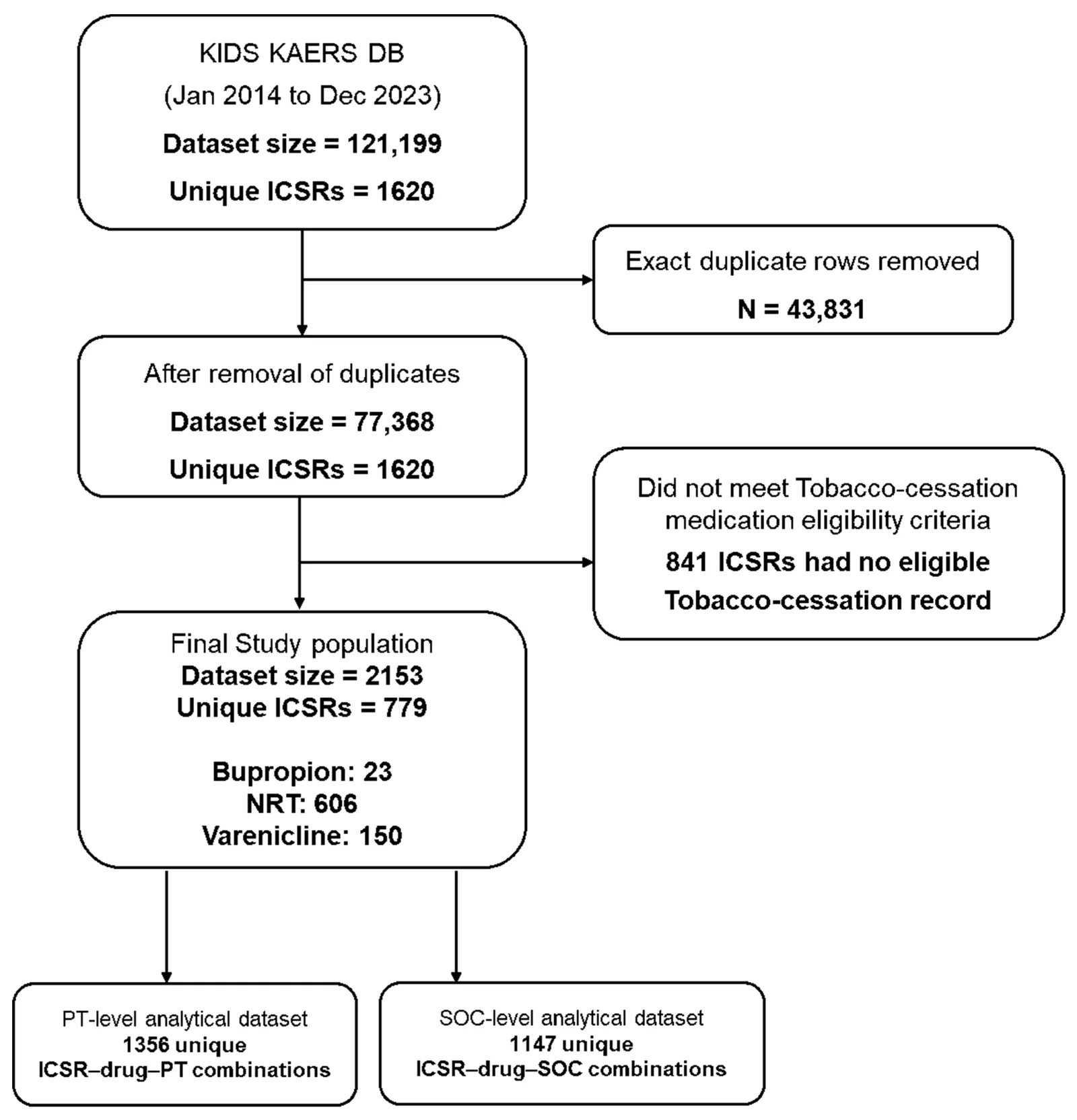
Flow diagram of data preprocessing and ICSR selection. Dataset size refers to the number of rows in the joined dataset after each data-processing step and does not necessarily represent unique drug–event relationships.

A total of 779 unique ICSRs involving tobacco cessation medications were included, comprising 1356 unique ICSR–drug–PT combinations and 1147 unique ICSR–drug–SOC combinations (Figure 1). Age information was available for 221 ICSRs and was missing for 558 ICSRs (71.63%) (Table 2). Among ICSRs with available age information, the median age was 47 years (IQR 29), and the 50–59-year age group was the most frequently represented (*n* = 45, 20.36%). Sixteen of the 221 ICSRs with available age information (7.24%) involved individuals aged <18 years. Sex information was available for 571 ICSRs, of which 458 (80.21%) involved male individuals. Reports from the general public constituted the largest primary reporter group (*n* = 341, 43.77%), followed by pharmacists (*n* = 251, 32.22%). NRT accounted for 606 ICSRs (77.79%), followed by varenicline (*n* = 150, 19.26%) and bupropion (*n* = 23, 2.95%). Forty-seven ICSRs (6.03%) involved at least one concomitant medication.

**Figure 2:**
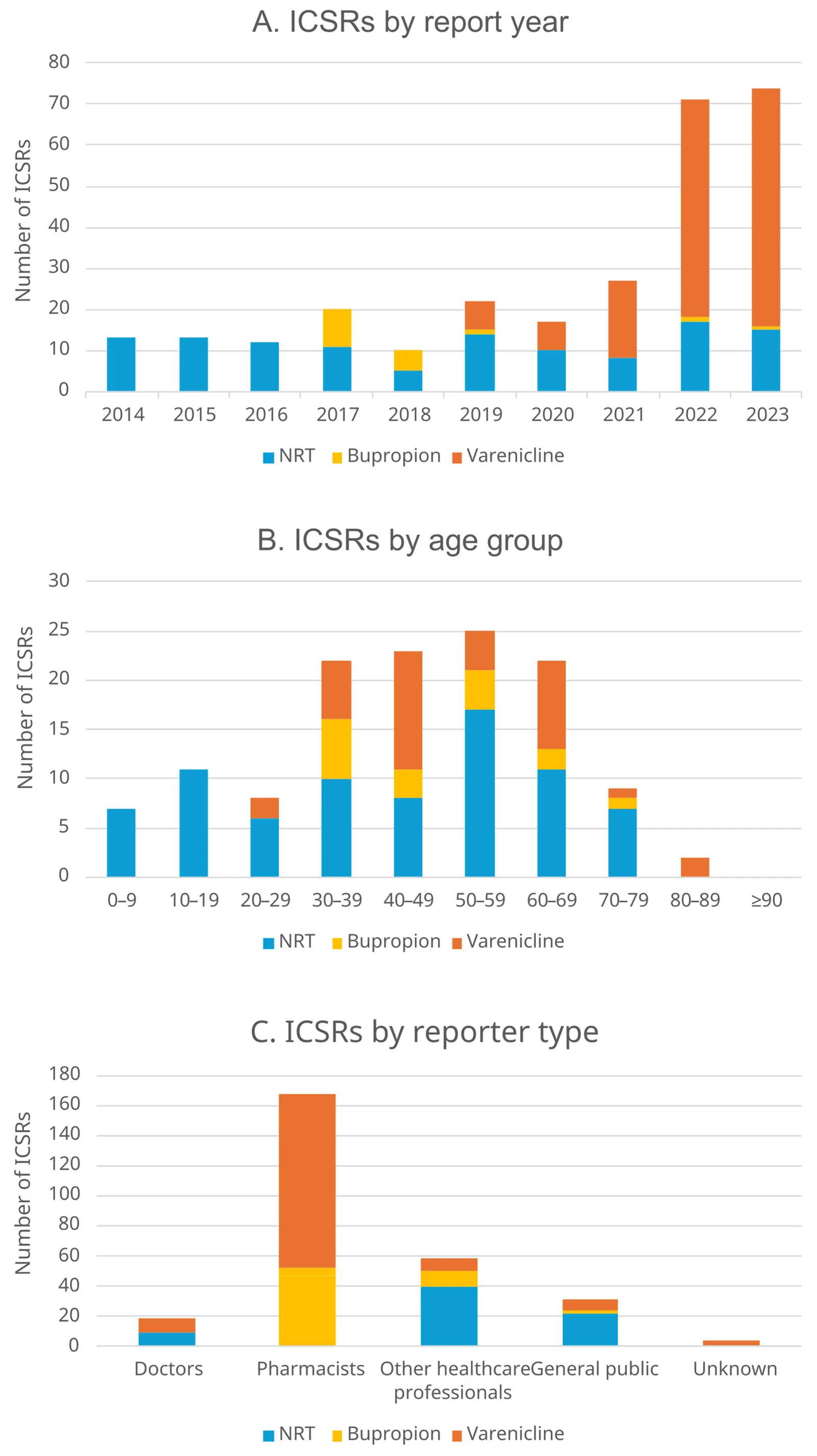
Distribution of included individual case safety reports (ICSRs) by report year, age group, and primary reporter type, stratified by tobacco-cessation medication. (**A**) ICSRs by report year, (**B**) ICSRs by age group, and (**C**) ICSRs by primary reporter type. Age-group analyses were restricted to ICSRs with available age information (*n* = 221). Age information was missing for 558 of 779 ICSRs (71.63%).

At the ICSR level, annual reporting patterns varied across the study period, with NRT accounting for most reports in earlier years and varenicline contributing a greater proportion of reports in 2022 and 2023 (Figure 2A). The 50–59-year age group had the largest number of reports; however, age-related patterns should be interpreted cautiously because age information was missing for 71.63% of the included ICSRs (Figure 2B). NRT was more frequently reported by the general public, whereas varenicline was relatively more common among pharmacist-reported ICSRs (Figure 2C). Regarding seriousness, only 52 serious ICSRs were identified (6.68%), comprising 49 NRT-related ICSRs and three bupropion-related ICSRs. No serious ICSRs were identified for varenicline (Table 3).

### 3.2. ADE Types and Signal Detection

Among the 779 included ICSRs, 52 serious ICSRs were identified. The most frequently reported serious criteria were other medically important events (*n* = 31, 59.62%) and hospitalization or prolonged hospitalization (*n* = 22, 42.31%) (Table 4). As ICSR could meet more than one seriousness criterion, these categories were not mutually exclusive.

**Table 4:** Seriousness criteria among serious ICSRs by tobacco-cessation medication.

Seriousness Criterion	Bupropion (*N* = 3)	NRT (*N* = 49)	Overall (*N* = 52)
Death	0 (0.00%)	2 (4.08%)	2 (3.85%)
Life-threatening	0 (0.00%)	1 (2.04%)	1 (1.92%)
Hospitalization/prolongation	0 (0.00%)	22 (44.90%)	22 (42.31%)
Disability/incapacity	0 (0.00%)	0 (0.00%)	0 (0.00%)
Congenital anomaly	0 (0.00%)	0 (0.00%)	0 (0.00%)
Other medically important event	3 (100.00%)	28 (57.14%)	31 (59.62%)

Seriousness criteria were not mutually exclusive. An ICSR could meet more than one seriousness criterion. Percentages were calculated using the number of serious ICSRs within each medication group as the denominator.

In the primary disproportionality analysis, SDRs were identified for psychiatric disorders with bupropion (PRR 3.42, ROR 6.56, χ^2^ =24.48, IC025 = 0.43), and for psychiatric disorders (PRR 2.86, ROR 3.97, χ^2^ = 49.05, IC025 = 0.56) and gastrointestinal system disorders (PRR 2.28, ROR 3.59, χ^2^ = 48.78, IC025 = 0.43) with varenicline (Table 5). No SDRs were identified for NRT. PT-level reporting frequencies based on unique ICSR–drug–WHO-ART PT combinations are presented descriptively in Table 6. In the PT-level disproportionality analysis, three SDRs were identified for bupropion: constipation (PRR 32.87; ROR 44.12; χ^2^= 94.15 IC025 = 0.86), anorexia (PRR 18.78, ROR 22.53, χ^2^ = 43.47, IC025 = 0.23), and insomnia (PRR 5.37, ROR 7.70, χ^2^ = 26.36, IC025 = 0.52) (Table 7). Five SDRs were identified for varenicline: malaise (PRR 5.48, ROR 6.06, χ^2^ 28.09, IC 1.37, IC025 0.50), dyspepsia (PRR 5.03, ROR 5.58, χ^2^ = 27.60, IC025 = 0.49), nausea (PRR 3.41, ROR 4.55, χ^2^ = 52.30, IC025 = 0.65), abnormal dreaming (PRR 146.03, ROR 165.04, χ^2^ = 72.88, IC025 = 1.03), and insomnia (PRR, 3.05, ROR 3.44, χ^2^ = 20.65, IC025 = 0.33). No PT-level SDRs met all predefined criteria for NRT. Although several PTs showed elevated PRR and ROR estimates for NRT, their IC025 values did not exceed zero and therefore did not satisfy the predefined SDR criteria.

### 3.3. Sensitivity Analysis Excluding ICSRs with Documented Age < 18 Years

In the sensitivity analysis excluding 16 ICSRs with documented age < 18 years while retaining ICSRs with missing age information, 763 ICSRs were included (Table 8 and Table 9). Considering the substantial proportion of reports with missing age information, this analysis was intended to assess the influence of reports with documented pediatric age rather than to represent an adult-only population. At the SOC level, SDRs remained detectable for psychiatric disorders with bupropion (PRR 3.35, ROR 6.40, χ^2^ = 23.65, IC025 = 0.40), and for psychiatric disorders (PRR 2.79, ROR 3.86, χ^2^ = 46.68, IC025 = 0.53) and gastro-intestinal system disorders (PRR 2.27, ROR 3.57, χ^2^ = 48.02, IC025 = 0.42) with varenicline. No SOC-level SDRs were identified for NRT (Table 8). In the PT-level sensitivity analysis, bupropion retained SDRs for constipation (PRR 32.17, ROR 43.18, χ^2^ = 92.06, IC025 = 0.85), anorexia (PRR 18.39, ROR 22.05, χ^2^ = 42.46, IC025 = 0.23), and insomnia (PRR 5.25, ROR 7.52, χ^2^ = 25.59, IC025 = 0.50). Varenicline retained SDRs for malaise (PRR 5.34, ROR 5.90, χ^2^ = 27.08, IC025 = 0.47), dyspepsia (PRR 5.25, ROR 5.83, χ^2^ = 28.31, IC025 = 0.50), nausea (PRR 3.44, ROR 4.59, χ^2^ = 52.33, IC025 = 0.64), abnormal dreaming (PRR 142.32, ROR 160.84, χ^2^ = 71.06, IC025 = 1.01), and insomnia (PRR 2.97, ROR 3.35, χ^2^ = 19.65, IC025 = 0.30) (Table 9). Overall, the detected SDRs remained consistent with those observed in the primary analysis.

In an additional sensitivity analysis restricted to drug–event relationships classified as certain, probable, or possible, 279 ICSRs were included. At the SOC level, SDRs were identified for NRT-related skin and appendage disorders (PRR 6.43, ROR 8.54, χ^2^ = 30.93, IC025 = 0.24) and application site disorders (PRR 50.37, ROR 59.46, χ^2^ 26.25, IC 1.08, IC025 0.15), as well as psychiatric disorders with varenicline (PRR 2.76, ROR 3.88, χ^2^ 21.83, IC 0.51, IC025 0.04) (Table S4). No SOC-level SDRs were identified for bupropion. At the PT level, NRT-related pruritus (PRR, 12.96; ROR, 15.26; χ^2^ = 21.60; IC025 = 0.08), rash (PRR, 25.92; ROR, 30.71; χ^2^ = 24.52; IC025 = 0.13), and application site reaction (PRR, 47.65; ROR, 55.69; χ^2^ = 24.70; IC025 = 0.12) met the predefined SDR criteria, whereas no PT-level SDRs were identified for bupropion or varenicline (Table S5). These findings indicate that the application of causality-based exclusion influenced the magnitude and classification of some disproportionality signals.

## 4. Discussion

This nationwide pharmacovigilance study characterized adverse event reporting patterns for NRT, bupropion, and varenicline using 779 unique ICSRs from the KAERS DB. In the primary ICSR-level analysis, SDRs were identified for psychiatric disorders with both bupropion and varenicline and for gastrointestinal disorders with varenicline, whereas no SDR met the predefined criteria for NRT. At the PT level, SDRs included constipation, anorexia, and insomnia with bupropion, and malaise, dyspepsia, nausea, abnormal dreaming, and insomnia with varenicline. Although application site reaction, pruritus, and rash were frequently reported with NRT, none met all predefined SDR criteria. A total of 52 ICSRs (6.68%) were classified as serious, including 49 involving NRT and three involving bupropion, while none were reported for varenicline. Two NRT-related ICSRs met the seriousness criterion of death, although their causal relationship with NRT remains uncertain. These findings are clinically relevant because all three medications are recommended for smoking cessation in current Korean clinical guidelines [27]. Nevertheless, as this study focused on signal detection rather than comparative risk estimation, the observed differences should be interpreted as relative reporting patterns rather than evidence of differences in medication safety.

The NRT findings showed reporting patterns broadly consistent with established formulation-related AE profiles. Although skin and appendages disorders and application site disorders did not meet all predefined SDR criteria, application site reaction, pruritus, and rash were frequently reported at the PT level. Previous evidence has consistently shown that NRT is associated with formulation-specific local AEs, including skin irritation with transdermal nicotine and oral or pharyngeal symptoms with orally administered formulations [28]. These findings support the clinical plausibility of the observed reporting patterns. However, standardized formulation and route information were not consistently available across the entire KAERS study period; therefore, nicotine-containing products were analyzed collectively as NRTs, and the observed reporting patterns cannot be attributed to a specific formulation. For application-site disorders, the absence of events in the comparator group results in very large PRR and ROR estimates, which should be interpreted with caution. Despite these elevated estimates, the corresponding IC025 values did not exceed zero, and the predefined SDR criteria were therefore not met.

A total of 52 serious ICSRs (6.68%) were identified in the final dataset, including 49 involving NRT and three involving bupropion, while none involved varenicline. Among these, two NRT-related ICSRs met the seriousness criterion of death. Although these reports are relevant for pharmacovigilance characterization, differences in serious ICSR counts do not allow direct comparisons of serious-event risk across medications. Serious adverse events associated with NRT have been uncommon in randomized studies, while its more established adverse effects are generally formulation-specific and non-serious [27,28]. In Korea, NRT may also be obtained as an over-the-counter medication and through public smoking-cessation programs, creating patterns of exposure and reporting that may differ from those of prescription-only therapies [27]. These differences may contribute to the distribution of spontaneous reports, although their magnitude cannot be evaluated without population-level utilization data. Thus, the observed distribution of serious ICSRs should be interpreted in the context of differences in medication utilization and reporting practices rather than as evidence of differential serious-event risk.

Varenicline showed SDRs for psychiatric and gastrointestinal disorders in the primary analysis. At the PT-level, insomnia and abnormal dreaming met the predefined SDR criteria, along with nausea, dyspepsia and malaise. These patterns are compatible with the established tolerability profile of varenicline. In the EAGLES randomized trial, varenicline was not associated with a significant increase in moderate-to-severe neuropsychiatric adverse events relative to nicotine patch or placebo [10]. Similarly, a meta-analysis of 39 randomized trials found no evidence of increased suicide, suicidal ideation, depression, or death, while showing higher frequencies of insomnia, sleep disorders, and abnormal dreams [29]. A nationwide Korean post-marketing surveillance study also reported nausea, abnormal dreams, insomnia, dyspepsia, and headache among the most commonly observed adverse events with varenicline [30]. The psychiatric and gastrointestinal SDRs observed in the present study are consistent with previously reported AE profiles and therefore should not be interpreted as evidence of increased serious neuropsychiatric or gastrointestinal risk.

Bupropion showed an SDR for psychiatric disorders at the SOC levels, while constipation, anorexia, and insomnia met the predefined SDR criteria at the PT level. However, only 23 bupropion ICSRs were available, substantially fewer than for NRT or varenicline, limiting the stability and precision of disproportionality estimation. Accordingly, these SDRs should be interpreted cautiously, particularly given the potential influence of small numbers of reports on disproportionality estimates. Previous studies have described established AE profiles for bupropion, including psychiatric and central nervous system events, and rare SAEs such as seizures have also been documented in post-marketing and regulatory safety data [31,32]. The psychiatric SDR observed in this study is broadly consistent with these established safety concerns, although no seizure-related SDR was detected. In a spontaneous reporting database, sparse reporting can substantially limit the ability to detect disproportionality, particularly when events are distributed across multiple PTs or SOCs. Furthermore, the identification of SDRs does not establish a causal relationship or indicate a higher incidence of AEs compared with other smoking-cessation medications. Further studies incorporating larger populations and comprehensive safety data are warranted to assess the robustness and clinical relevance of the identified SDRs for bupropion.

The sensitivity analysis with causality-based restriction further demonstrated the influence of report selection on signal detection. When the causality-based exclusion criteria were applied, 279 ICSRs were retained, and the pattern of detected SDRs differed from that observed in the primary analysis. At the SOC level, SDRs were identified for skin and appendages disorders and application site disorders with NRT and for psychiatric disorders with varenicline, whereas no SDRs were identified for bupropion. At the PT level, pruritus, rash, and application site reaction met the predefined SDR criteria for NRT, whereas no PT-level SDRs were identified for bupropion or varenicline. These findings demonstrate that the detection and classification of SDRs were sensitive to the application of causality-based report selection. This variability implies the influence of report-selection criteria on signal detection and emphasizes the importance of cautious interpretation of disproportionality findings from spontaneous reporting data.

Age-related findings warrant cautious interpretation. Among the 221 ICSRs with available age information, reports were broadly distributed across individuals aged 20–69 years, with the 50–59-year group being numerically the most frequently represented but without a marked concentration in any single age group. Age information was missing for 558 of the 779 ICSRs (71.63%), further limiting interpretation of age-related reporting patterns. Sixteen ICSRs with documented age involved individuals aged <18 years, all of which involved NRT. In the sensitivity analysis excluding these 16 ICSRs while retaining those with missing age information, 763 ICSRs remained, and all SOC- and PT-level SDRs identified in the primary analysis were retained. These findings suggest that the detected SDR patterns were not materially altered by the exclusion of reports with documented pediatric age. Nevertheless, the substantial proportion of missing age information precludes definitive age-specific interpretation. Further studies using more complete age information and larger populations are warranted to evaluate potential age-related differences in AE reporting patterns associated with smoking-cessation medications.

The temporal reporting pattern also warrants cautious interpretation. Changes in reporting patterns during 2021–2023 should be considered in the context of changes in the national AE reporting system. This period overlapped with the ongoing transition to an ICH E2B(R3)-based electronic adverse-event reporting system, which began before 2022 and culminated in the discontinuation of KAERS-KIDS and unification under KAERS-NeDrug in March 2023 [21,33,34]. Regulatory attention to varenicline also increased in 2021 following the detection of N-nitroso-varenicline and subsequent safety-management measures [35,36]. These changes may have affected reporting practices and reporting opportunities, including the possibility of stimulated reporting. Although the contribution of these systems and regulatory changes cannot be quantified from the present data, they provide important context for temporal reporting patterns observed in this study. Accordingly, temporal variations in reporting frequency should not be interpreted as evidence of a corresponding increase in the incidence or clinical risk of AEs.

The general public represented the largest proportion of primary reporters, accounting for 341 ICSRs (43.77%), followed by pharmacists (*n* = 251, 32.22%). This distribution emphasizes the contributions of both public and healthcare professionals in pharmacovigilance surveillance of smoking-cessation medications. The relatively high proportion of reports submitted by the general public may partly reflect the accessibility of NRT as an over-the-counter medication and its availability through national smoking-cessation programs in Korea, which may facilitate direct reporting of suspected AEs by individuals [27]. Moreover, the transition to KAERS-NeDrug during the study period may have influenced reporting pathways and the composition of primary reporters, although its contribution to the observed distribution cannot be determined from the available data. Among healthcare professionals, pharmacists accounted for a considerable proportion of reports on smoking-cessation medications [37]. Their frequent contact with patients during medication dispensing and counseling may provide opportunities to identify and report suspected AEs, thereby contributing to medication safety surveillance. Pharmacovigilance is an integral component of patient safety, and systematic AE reporting by both healthcare professionals and patients provides information that can support safer medication use and continuous improvement in the quality of care [38,39,40,41]. However, the observed distribution of reports across reporter groups in this study should not be interpreted as a measure of reporting quality or relative clinical contribution, because the number of reporting opportunities for each group was unavailable.

From a patient-safety perspective, this study provides pharmacovigilance evidence on spontaneous AE reporting patterns associated with three tobacco cessation medications in Korea. The ICSR-level approach reduced potential inflation from repeated counting of multiple PTs within the same SOC from a single ICSR, while the predefined SDR criteria incorporated both frequentist and Bayesian measures. As an additional methodological check, we also examined whether SDR classification changed when the Bayesian component was recalculated using an alternative Gamma–Poisson shrinkage approach (Table S6) [42]. The three SOC-level SDRs identified in the primary BCPNN analysis remained detectable, while application site disorders with NRT additionally met the predefined SDR criteria under the Gamma–Poisson approach [38,39]. These findings suggest that SDR detection may be influenced by the choice of Bayesian shrinkage method, although the primary signals remained consistent across both approaches. Early identification and continued monitoring of medication-related safety concerns through national pharmacovigilance can complement evidence from clinical trials by detecting rare, unexpected, or population-specific adverse events that may not be fully characterized before medication approval. Spontaneous reporting systems provide an important source of post-marketing safety information that can support signal detection, further safety assessment, and regulatory or clinical risk minimization measures [13,38,39]. These activities contribute to medication safety by allowing emerging safety concerns to be recognized and evaluated throughout the medication life cycle, thereby supporting safer medication use and the quality of care [38,39]. Moreover, pharmacovigilance findings are not intended to replace evidence from clinical trials or pharmacoepidemiologic studies; rather, they complement these data sources by identifying safety signals that can subsequently be evaluated using longitudinal healthcare data [13]. Thus, systematic pharmacovigilance provides a continuous link between real-world AE reporting, signal detection, and subsequent medication-safety surveillance, supporting its role as an essential component of patient safety and healthcare quality.

This study has several limitations that should be acknowledged. First, this study was based on a spontaneous reporting pharmacovigilance database, which is inherently subject to underreporting, reporting bias, and incomplete clinical information. Spontaneous reporting systems are also susceptible to stimulated reporting, notoriety bias, and differential reporting across medications and reporter groups, which may influence the observed reporting patterns independently of the underlying safety profile. Additionally, changes in AE reporting system and regulatory attention during the study period may have influenced temporal reporting patterns, although their effects could not be quantified. Second, disproportionality analysis is designed to detect potential SDRs but cannot establish causal relationships between tobacco cessation medications and AEs. Consistent with the READUS-PV recommendations, the findings should be interpreted as signals of disproportionate reporting rather than evidence of causal association or comparative safety. As population-level exposure denominators were unavailable, the analyses cannot estimate AE incidence or absolute or relative risks. Furthermore, as the available KAERS data was restricted to reports involving three prespecified smoking-cessation medications, the entire KAERS database could not be used as the comparator. The identified SDRs therefore represent relative reporting patterns among NRT, bupropion, and varenicline and may be affected by competition or masking within this restricted comparator. Furthermore, causality-based restriction reduced the dataset from 779 to 279 ICSRs and altered the detected SDR patterns, implying the influence of report selection on signal detection. Moreover, 52 serious ICSRs were identified, but the small number of serious reports for bupropion and the absence of such reports for varenicline limited meaningful assessment of medication-specific serious-event patterns or factors associated with seriousness. Third, residual confounding could not be fully addressed due to missing clinically important variables, including smoking history, nicotine dependence severity, underlying comorbidities, and concomitant medications. Confounding by indication may therefore have influenced the observed reporting patterns. Fourth, detailed treatment-related information including dosage, duration of therapy, NRT formulation, route of administration, and medication adherence was not consistently available. These factors may influence the occurrence and severity of AEs, particularly for NRT, where different formulations and dosing strategies are commonly used. The lack of standardized formulation information also prevented attribution of the observed NRT-related reporting patterns to specific formulations. Additionally, information regarding pregnancy status or pregnancy planning was not available in the KIDS KAERS DB. Therefore, we were unable to evaluate AE patterns among pregnant women or women who were planning pregnancy. Fifth, missing demographic and clinical information limited subgroup-specific interpretation. Age information was unavailable for 558 ICSRs (71.63%), limiting age-specific interpretation. In the sensitivity analysis excluding 16 ICSRs involving individuals aged < 18 years, all SDRs identified in the primary analysis remained detectable. However, the substantial proportion of missing age information limits the definitive age-specific interpretations. Thus, these findings warrant cautious interpretation of age-related reporting patterns. Furthermore, the limited clinical information made it difficult to determine whether reports involving individuals aged < 18 years reflected therapeutic use, off-label use, inappropriate dosing or accidental exposure. Because the study was based on a single national pharmacovigilance system, the findings may also have limited generalizability to healthcare systems with different prescribing practices, tobacco control policies, and pharmacovigilance structures. Despite these limitations, this study has several strengths. This study characterized post-marketing AE reporting patterns. A methodological strength of this study was the use of the ICSR as the reporting unit, with each unique ICSR–drug–PT and ICSR–drug–SOC combination counted once. This approach reduced the potential for inflation of SOC-level counts when multiple PTs belonging to the same SOC were reported within a single ICSR. Signal identification was based on predefined criteria requiring concordance across frequentist and Bayesian disproportionality measures rather than reliance on a single measure, providing a more stringent framework for identifying SDRs. The sensitivity of SDR classification to the choice of Bayesian shrinkage method was further assessed using an alternative approach. Furthermore, the study was designed and reported in accordance with the READUS-PV recommendations, enhancing the transparency, consistency, and interpretability of the disproportionality analyses. Collectively, these features enhance the relevance of the findings for ongoing pharmacovigilance surveillance and provide a basis for further evaluation in pharmacoepidemiologic investigations.

## 5. Conclusions

This nationwide pharmacovigilance study characterized spontaneous AE reporting patterns associated with NRT, bupropion, and varenicline in Korea using 779 unique ICSRs. SOC-level disproportionality analysis identified SDRs for psychiatric disorders with bupropion and for psychiatric and gastrointestinal disorders with varenicline, whereas no SDR was identified for NRT. At the PT level, SDRs were identified for constipation, anorexia, and insomnia with bupropion and for malaise, dyspepsia, nausea, abnormal dreaming, and insomnia with varenicline. A total of 52 serious ICSRs (6.68%) were identified, including 49 ICSRs involving NRT and 3 involving bupropion. All SDRs identified in the primary analysis remained detectable after excluding 16 ICSRs involving individuals aged < 18 years. However, causality-based restriction altered the detected SDR patterns, implying the influence of report selection on SDR detection. These findings should be interpreted as relative reporting patterns rather than estimates of incidence, clinical risk, or causal association. Overall, the results provide pharmacovigilance evidence that may help prioritize specific medication–event patterns for further evaluation and support medication-safety surveillance in clinical practice. Further studies with appropriate exposure denominators and more detailed clinical information are warranted to confirm and characterize these reporting patterns.

## Figures and Tables

**Table 1:** Definition and signal detection criteria of data-mining indices.

Signal Index	Definition	Criteria of Signal
PRR	$\frac{A/(A+B)}{C/(C+D)}$	PRR ≥ 2
ROR	$\frac{A/B}{C/D}$	ROR ≥ 2
Pearson’s $\chi^{2}$	$\chi^{2}=\frac{{N(AD-BC)}^{2}}{\left(A+B)(C+D)(A+C)(B+D\right)}$	$\chi^{2}\geq$ 4
IC	${log}_{2}\frac{P(SOC,\;drug)}{P\left(SOC\right)\times P(drug)}$ ${IC}_{025}$ = E(IC) *−* 2√V(IC)	${IC}_{025}$ > 0
	Specific SOC	All other SOC
Specific drugs	A	B
Other tobacco cessation medications	C	D

N = A + B + C + D. A represents the number of ICSRs involving the target medication and the specific SOC; B, the target medication and all other SOCs; C, either of the other two smoking-cessation medications and the specific SOC; and D, either of the other two smoking-cessation medications and all other SOCs. An SDR was defined when all of the following criteria were met: A ≥ 3, PRR ≥ 2, ROR ≥ 2, Pearson’s χ^2^ ≥ 4, and IC025 > 0.

**Table 2:** Demographic and clinical characteristics of the included ICSRs (*N* = 779).

Characteristics	No. of ICSRs (%) or Median (IQR)
**Age (years) ^a^**	**Median: 47; IQR: 29**
0–9	9 (4.07%)
10–19	11 (4.98%)
20–29	29 (13.12%)
30–39	32 (14.48%)
40–49	39 (17.65%)
50–59	45 (20.36%)
60–69	39 (17.65%)
70–79	14 (6.33%)
80–89	3 (1.36%)
Missing ^c^	558 (71.63%)
**Sex ^b^**	
Men	458 (80.21%)
Women	113 (19.79%)
Missing ^c^	208 (26.70%)
**Causality ^d^**	
Certain	10 (1.28%)
Probable/Likely	84 (10.78%)
Possible	187 (24.01%)
Unlikely	6 (0.77%)
Conditional/Unclassified	97 (12.45%)
Unassessable/Unclassifiable	24 (3.08%)
Missing/Not recorded	395 (50.71%)
**Seriousness**	
Serious ICSR	52 (6.68%)
Non-serious ICSR	727 (93.32%)
**Primary reporter type**	
Doctors	53 (6.80%)
Pharmacists	251 (32.22%)
Other healthcare professionals	73 (9.37%)
General Public	341 (43.77%)
Unknown	61 (7.83%)
**Anti-smoking medication types**	
Bupropion	23 (2.95%)
NRT	606 (77.79%)
Varenicline	150 (19.26%)
**Types of concomitantly used medication ^e^**	
Any concomitant medication	47 (6.03)
Other drugs	26 (3.34)
Gastrointestinal drugs	23 (2.95)
Cardiovascular drugs	22 (2.82)
Antipsychotics	17 (2.18)
Anxiolytics	16 (2.05)
Antihyperlipidemics	13 (1.67)
Analgesics	13 (1.67)
Anticonvulsants	12 (1.54)
Hypnotics	11 (1.41)
Antibiotics	10 (1.28)
Anticoagulants	8 (1.03)
Respiratory drugs	8 (1.03)
Antidepressants	7 (0.90)
Antiparkinsonian drugs	7 (0.90)
Antihistamines	7 (0.90)
Diuretics	6 (0.77)
Antineoplastic agents	2 (0.26)
Antidiabetic drugs	1 (0.13)

Abbreviation: IQR: interquartile range. Values are presented as *n* (%) unless otherwise indicated. Concomitant medication categories were not mutually exclusive; an ICSR could be included in more than one category. ^a^ Percentages for age categories were calculated among ICSRs with available age information (*n* = 221). Among ICSRs with available age information, 16 (7.24%) involved individuals aged < 18 years. ^b^ Percentages for sex categories were calculated among ICSRs with available sex information (*n* = 571). ^c^ Percentages for missing age and sex were calculated using all included ICSRs (*n* = 779). ^d^ Causality categories were not mutually exclusive because a single ICSR could contain multiple drug–event relationships with different recorded causality assessments. Each ICSR was counted once within each applicable causality category; therefore, percentages do not sum to 100%. Percentages were calculated using all included ICSRs (*N* = 779). ^e^ Multiple concomitant medication categories could be reported within a single ICSR, and therefore, percentages do not sum to 100%.

**Table 3:** Distribution of serious and non-serious ICSRs by smoking-cessation medication.

Tobacco Cessation Medications	Serious ICSR, *n* (%)	Non-Serious ICSR, *n* (%)	Total, *n* (%)
NRT	49 (8.09%)	557 (91.91%)	606 (77.79%)
Bupropion	3 (13.04%)	20 (86.96%)	23 (2.95%)
Varenicline	0 (0.00%)	150 (100.00%)	150 (19.26%)
Overall	52 (6.68%)	727 (93.32%)	779 (100.00%)

Percentages in the serious and non-serious columns were calculated within each smoking-cessation medication group. Percentages in the Total column were calculated using all included ICSRs (*N* = 779). A serious ICSR was defined as an ICSR containing at least one serious adverse event.

**Table 5:** SOC-level disproportionality analysis of smoking-cessation medications without causality-based exclusion (*n* = 779 ICSRs).

Medication	WHO-ART SOC	Target, *n*/*N* (%)	Comparator, *n*/*N* (%)	PRR (95% CI)	ROR (95% CI)	χ^2^	IC	IC025	IC975
Bupropion	Skin and appendages disorders	5/23 (21.74%)	142/756 (18.78%)	1.16 (0.53–2.55)	1.20 (0.44–3.29)	0.13	0.12	−1.21	1.44
	Central & peripheral nervous system disorders	5/23 (21.74%)	128/756 (16.93%)	1.28 (0.58–2.83)	1.36 (0.50–3.74)	0.36	0.23	−1.1	1.56
	Psychiatric disorders	13/23 (56.52%)	125/756 (16.53%)	3.42 (2.31–5.06)	6.56 (2.81–15.30)	24.48	1.41	0.43	2.4
	Gastro-intestinal system disorders	10/23 (43.48%)	206/756 (27.25%)	1.60 (0.99–2.58)	2.05 (0.89–4.76)	2.93	0.52	−0.53	1.58
	Respiratory system disorders	3/23 (13.04%)	46/756 (6.08%)	2.14 (0.72–6.39)	2.32 (0.66–8.08)	1.83	0.66	−0.94	2.26
	Body as a whole-general disorders	5/23 (21.74%)	122/756 (16.14%)	1.35 (0.61–2.97)	1.44 (0.53–3.96)	0.51	0.29	−1.04	1.61
NRT	Skin and appendages disorders	135/606 (22.28%)	12/173 (6.94%)	3.21 (1.82–5.65)	3.85 (2.08–7.13)	20.69	0.23	−0.08	0.55
	Musculo-skeletal system disorders	19/606 (3.14%)	3/173 (1.73%)	1.81 (0.54–6.04)	1.83 (0.54–6.27)	0.96	0.09	−0.78	0.96
	Central & peripheral nervous system disorders	102/606 (16.83%)	31/173 (17.92%)	0.94 (0.65–1.35)	0.93 (0.60–1.44)	0.11	−0.03	−0.38	0.33
	Vision disorders	11/606 (1.82%)	0/173 (0.00%)	6.59 (0.39–111.33)	6.70 (0.39–114.30)	3.19	0.22	−0.95	1.39
	Hearing and vestibular disorders	7/606 (1.16%)	2/173 (1.16%)	1.00 (0.21–4.77)	1.00 (0.21–4.85)	0	−0.13	−1.49	1.23
	Special senses other, disorders	6/606 (0.99%)	0/173 (0.00%)	3.73 (0.21–65.83)	3.76 (0.21–67.01)	1.73	0.12	−1.41	1.66
	Psychiatric disorders	69/606 (11.39%)	69/173 (39.88%)	0.29 (0.21–0.38)	0.19 (0.13–0.29)	74.98	−0.64	−1.04	−0.24
	Gastro-intestinal system disorders	130/606 (21.45%)	86/173 (49.71%)	0.43 (0.35–0.53)	0.28 (0.19–0.39)	53.63	−0.37	−0.66	−0.08
	Liver and biliary system disorders	3/606 (0.50%)	0/173 (0.00%)	2.01 (0.10–38.66)	2.01 (0.10–39.15)	0.86	−0.04	−2.07	2
	Metabolic and nutritional disorders	22/606 (3.63%)	4/173 (2.31%)	1.57 (0.55–4.50)	1.59 (0.54–4.68)	0.72	0.07	−0.74	0.88
	Cardiovascular disorders, general	14/606 (2.31%)	5/173 (2.89%)	0.80 (0.29–2.19)	0.79 (0.28–2.24)	0.19	−0.14	−1.11	0.84
	Heart rate and rhythm disorders	19/606 (3.14%)	2/173 (1.16%)	2.71 (0.64–11.53)	2.77 (0.64–12.00)	2.01	0.15	−0.73	1.03
	Vascular (extracardiac) disorders	11/606 (1.82%)	0/173 (0.00%)	6.59 (0.39–111.33)	6.70 (0.39–114.30)	3.19	0.22	−0.95	1.39
	Respiratory system disorders	41/606 (6.77%)	8/173 (4.62%)	1.46 (0.70–3.06)	1.50 (0.69–3.26)	1.05	0.08	−0.51	0.67
	Red blood cell disorders	3/606 (0.50%)	0/173 (0.00%)	2.01 (0.10–38.66)	2.01 (0.10–39.15)	0.86	−0.04	−2.07	2
	White cell and RES disorders	6/606 (0.99%)	0/173 (0.00%)	3.73 (0.21–65.83)	3.76 (0.21–67.01)	1.73	0.12	−1.41	1.66
	Platelet, bleeding & clotting disorders	4/606 (0.66%)	0/173 (0.00%)	2.58 (0.14–47.69)	2.59 (0.14–48.38)	1.15	0.04	−1.78	1.86
	Urinary system disorders	4/606 (0.66%)	4/173 (2.31%)	0.29 (0.07–1.13)	0.28 (0.07–1.13)	3.61	−0.67	−2.28	0.93
	Reproductive disorders, female	3/606 (0.50%)	0/173 (0.00%)	2.01 (0.10–38.66)	2.01 (0.10–39.15)	0.86	−0.04	−2.07	2
	Body as a whole-general disorders	96/606 (15.84%)	31/173 (17.92%)	0.88 (0.61–1.28)	0.86 (0.55–1.35)	0.43	−0.05	−0.41	0.32
	Application site disorders	111/606 (18.32%)	0/173 (0.00%)	63.92 (3.99–1023.16)	78.08 (4.83–1262.97)	36.95	0.35	−0.01	0.71
	Secondary terms-events	67/606 (11.06%)	1/173 (0.58%)	19.13 (2.67–136.79)	21.38 (2.95–155.17)	18.55	0.32	−0.15	0.79
Varenicline	Skin and appendages disorders	7/150 (4.67%)	140/629 (22.26%)	0.21 (0.10–0.44)	0.17 (0.08–0.37)	24.48	−1.88	−2.94	−0.83
	Central & peripheral nervous system disorders	26/150 (17.33%)	107/629 (17.01%)	1.02 (0.69–1.50)	1.02 (0.64–1.64)	0.01	0.01	−0.62	0.64
	Psychiatric disorders	56/150 (37.33%)	82/629 (13.04%)	2.86 (2.14–3.82)	3.97 (2.65–5.95)	49.05	1.04	0.56	1.51
	Gastro-intestinal system disorders	76/150 (50.67%)	140/629 (22.26%)	2.28 (1.84–2.82)	3.59 (2.47–5.20)	48.78	0.85	0.43	1.26
	Metabolic and nutritional disorders	3/150 (2.00%)	23/629 (3.66%)	0.55 (0.17–1.80)	0.54 (0.16–1.82)	1.03	−0.63	−2.19	0.92
	Cardiovascular disorders, general	5/150 (3.33%)	14/629 (2.23%)	1.50 (0.55–4.09)	1.51 (0.54–4.27)	0.62	0.3	−1.05	1.66
	Respiratory system disorders	5/150 (3.33%)	44/629 (7.00%)	0.48 (0.19–1.18)	0.46 (0.18–1.18)	2.76	−0.83	−2.08	0.43
	Urinary system disorders	4/150 (2.67%)	4/629 (0.64%)	4.19 (1.06–16.58)	4.28 (1.06–17.32)	4.91	0.87	−0.75	2.49
	Body as a whole-general disorders	26/150 (17.33%)	101/629 (16.06%)	1.08 (0.73–1.60)	1.10 (0.68–1.76)	0.14	0.07	−0.56	0.7

Abbreviation: RES: reticuloendothelial system.

**Table 6:** Frequently reported WHO-ART preferred terms by tobacco-cessation medication.

WHO-ART SOC	WHO-ART PT	Bupropion, *n* (%) *N* = 23	NRT, *n* (%) *N* = 606	Varenicline, *n* (%) *N* = 150
**Skin** **and appendage disorders**	Bullous eruption	0 (0.00%)	13 (2.15%)	0 (0.00%)
	Dermatitis contact	3 (13.04%)	2 (0.33%)	0 (0.00%)
	Livedo reticularis	0 (0.00%)	3 (0.50%)	0 (0.00%)
	Pruritus	0 (0.00%)	48 (7.92%)	3 (2.00%)
	Rash	0 (0.00%)	47 (7.76%)	1 (0.67%)
	Rash erythematous	0 (0.00%)	11 (1.82%)	0 (0.00%)
	Skin discoloration	0 (0.00%)	5 (0.83%)	0 (0.00%)
	Skin disorder	0 (0.00%)	6 (0.99%)	0 (0.00%)
	Skin dry	0 (0.00%)	3 (0.50%)	0 (0.00%)
	Sweating increased	0 (0.00%)	3 (0.50%)	2 (1.33%)
	Urticaria	1 (4.35%)	19 (3.14%)	1 (0.67%)
**Musculo-skeletal system disorders**	Arthralgia	0 (0.00%)	3 (0.50%)	0 (0.00%)
	Myalgia	0 (0.00%)	11 (1.82%)	1 (0.67%)
	Skeletal pain	1 (4.35%)	6 (0.99%)	0 (0.00%)
**Central & peripheral nervous system disorders**	Convulsions grand mal	0 (0.00%)	3 (0.50%)	0 (0.00%)
	Dizziness	1 (4.35%)	59 (9.74%)	12 (8.00%)
	Headache	3 (13.04%)	34 (5.61%)	10 (6.67%)
	Vertigo	0 (0.00%)	4 (0.66%)	0 (0.00%)
**Vision disorders**	Diplopia	0 (0.00%)	5 (0.83%)	0 (0.00%)
	Vision abnormal	0 (0.00%)	4 (0.66%)	0 (0.00%)
**Hearing and vestibular disorders**	Application site pain	0 (0.00%)	6 (0.99%)	0 (0.00%)
**Special senses other, disorders**	Taste perversion	0 (0.00%)	5 (0.83%)	0 (0.00%)
**Psychiatric disorders**	Aggressive reaction	0 (0.00%)	5 (0.83%)	1 (0.67%)
	Anorexia	4 (17.39%)	1 (0.17%)	6 (4.00%)
	Anxiety	0 (0.00%)	9 (1.49%)	1 (0.67%)
	Apathy	0 (0.00%)	1 (0.17%)	4 (2.67%)
	Delirium	3 (13.04%)	0 (0.00%)	0 (0.00%)
	Depression	2 (8.70%)	2 (0.33%)	3 (2.00%)
	Dreaming abnormal	0 (0.00%)	0 (0.00%)	17 (11.33%)
	Drug dependence	0 (0.00%)	19 (3.14%)	0 (0.00%)
	Hallucination	0 (0.00%)	4 (0.66%)	1 (0.67%)
	Insomnia	8 (34.78%)	25 (4.13%)	24 (16.00%)
	Paroniria	1 (4.35%)	4 (0.66%)	2 (1.33%)
	Sleep disorder	0 (0.00%)	4 (0.66%)	6 (4.00%)
	Somnolence	1 (4.35%)	4 (0.66%)	4 (2.67%)
	Suicide attempt	0 (0.00%)	4 (0.66%)	0 (0.00%)
**Gastro-intestinal system disorders**	Abdominal pain	1 (4.35%)	17 (2.81%)	3 (2.00%)
	Constipation	6 (26.09%)	4 (0.66%)	2 (1.33%)
	Diarrhoea	1 (4.35%)	3 (0.50%)	3 (2.00%)
	Dyspepsia	1 (4.35%)	14 (2.31%)	18 (12.00%)
	Glossitis	0 (0.00%)	4 (0.66%)	0 (0.00%)
	Gum hyperplasia	0 (0.00%)	3 (0.50%)	0 (0.00%)
	Hiccup	0 (0.00%)	8 (1.32%)	0 (0.00%)
	Mouth dry	1 (4.35%)	3 (0.50%)	3 (2.00%)
	Nausea	3 (13.04%)	56 (9.24%)	48 (32.00%)
	Stomatitis	0 (0.00%)	4 (0.66%)	0 (0.00%)
	Stomatitis ulcerative	0 (0.00%)	4 (0.66%)	0 (0.00%)
	Tooth disorder	0 (0.00%)	5 (0.83%)	0 (0.00%)
	Vomiting	0 (0.00%)	32 (5.28%)	12 (8.00%)
**Liver and biliary system disorders**	Hepatic function abnormal	0 (0.00%)	3 (0.50%)	0 (0.00%)
**Metabolic and nutritional** **disorders**	Diabetes mellitus	0 (0.00%)	3 (0.50%)	0 (0.00%)
	Hyperglycaemia	0 (0.00%)	11 (1.82%)	0 (0.00%)
	Xerophthalmia	0 (0.00%)	3 (0.50%)	1 (0.67%)
**Cardiovascular disorders, general**	Hypertension	0 (0.00%)	10 (1.65%)	2 (1.33%)
	Hypotension	0 (0.00%)	2 (0.33%)	3 (2.00%)
**Heart rate and rhythm disorders**	Palpitation	0 (0.00%)	16 (2.64%)	1 (0.67%)
	Tachycardia	1 (4.35%)	3 (0.50%)	0 (0.00%)
**Vascular (extracardiac) disorders**	Cerebral haemorrhage	0 (0.00%)	5 (0.83%)	0 (0.00%)
	Flushing	0 (0.00%)	4 (0.66%)	0 (0.00%)
**Respiratory system disorders**	Coughing	3 (13.04%)	4 (0.66%)	3 (2.00%)
	Dyspnoea	0 (0.00%)	8 (1.32%)	0 (0.00%)
	Laryngitis	0 (0.00%)	8 (1.32%)	0 (0.00%)
	Pharyngitis	0 (0.00%)	14 (2.31%)	0 (0.00%)
	Pneumonitis	0 (0.00%)	3 (0.50%)	0 (0.00%)
	Sputum increased	0 (0.00%)	2 (0.33%)	3 (2.00%)
**Red blood cell disorders**	Anaemia	0 (0.00%)	3 (0.50%)	0 (0.00%)
**White cell and RES disorders**	Lymphadenopathy	0 (0.00%)	6 (0.99%)	0 (0.00%)
**Body as a whole-general** **disorders**	Allergic reaction	0 (0.00%)	3 (0.50%)	0 (0.00%)
	Asthenia	0 (0.00%)	3 (0.50%)	1 (0.67%)
	Chest pain	0 (0.00%)	20 (3.30%)	4 (2.67%)
	Fatigue	0 (0.00%)	1 (0.17%)	3 (2.00%)
	Fever	0 (0.00%)	3 (0.50%)	0 (0.00%)
	Leg pain	0 (0.00%)	5 (0.83%)	0 (0.00%)
	Malaise	3 (13.04%)	10 (1.65%)	17 (11.33%)
	Medicine ineffective	0 (0.00%)	21 (3.47%)	0 (0.00%)
	Oedema	0 (0.00%)	9 (1.49%)	0 (0.00%)
	Pain	0 (0.00%)	4 (0.66%)	0 (0.00%)
	Temperature changed sensation	0 (0.00%)	5 (0.83%)	0 (0.00%)
	Withdrawal syndrome	0 (0.00%)	4 (0.66%)	0 (0.00%)
**Application site disorders**	Application site oedema	0 (0.00%)	12 (1.98%)	0 (0.00%)
	Application site reaction	0 (0.00%)	106 (17.49%)	0 (0.00%)
**Secondary terms-events**	Drug administration error	1 (4.35%)	6 (0.99%)	0 (0.00%)
	Drug quality problem	1 (4.35%)	12 (1.98%)	0 (0.00%)
	Inappropriate schedule of drug administration	0 (0.00%)	8 (1.32%)	0 (0.00%)
	Incorrect dose administered	0 (0.00%)	3 (0.50%)	0 (0.00%)
	Incorrect drug administration duration	0 (0.00%)	11 (1.82%)	0 (0.00%)
	Incorrect technique in drug usage process	0 (0.00%)	20 (3.30%)	0 (0.00%)
	Medication error	1 (4.35%)	9 (1.49%)	0 (0.00%)

Values are presented as *n* (%). Frequencies were calculated using unique ICSR–drug–WHO-ART PT combinations. Therefore, the same PT reported multiple times within the same ICSR for the same medication was counted once. PTs reported in at least three ICSRs for any one of the three smoking-cessation medications were included in the table. Percentages were calculated using the total number of ICSRs for each medication as the denominator. Abbreviation: RES: reticuloendothelial system.

**Table 7:** PT-level disproportionality analysis of smoking-cessation medications without causality-based exclusion (*n* = 779 ICSRs).

Medication	Preferred Term (PT)	Target, *n*/*N* (%)	Comparator, *n*/*N* (%)	PRR (95% CI)	ROR (95% CI)	χ^2^	IC	IC025	IC975
Bupropion	APPLICATION SITE REACTION	0/23 (0.00%)	106/756 (14.02%)	0.15 (0.01–2.31)	0.13 (0.01–2.16)	3.73	−2.1	−5.05	0.85
	CHEST PAIN	0/23 (0.00%)	24/756 (3.17%)	0.64 (0.04–10.28)	0.64 (0.04–10.78)	0.75	−0.82	−3.82	2.17
	MALAISE	3/23 (13.04%)	27/756 (3.57%)	3.65 (1.19–11.17)	4.05 (1.13–14.46)	5.41	1.04	−0.6	2.67
	HYPERTENSION	0/23 (0.00%)	12/756 (1.59%)	1.26 (0.08–20.69)	1.27 (0.07–22.05)	0.37	−0.48	−3.53	2.56
	DIZZINESS	1/23 (4.35%)	71/756 (9.39%)	0.46 (0.07–3.19)	0.44 (0.06–3.30)	0.68	−0.69	−2.84	1.45
	HEADACHE	3/23 (13.04%)	44/756 (5.82%)	2.24 (0.75–6.69)	2.43 (0.69–8.48)	2.05	0.69	−0.91	2.3
	ABDOMINAL PAIN	1/23 (4.35%)	20/756 (2.65%)	1.64 (0.23–11.73)	1.67 (0.21–13.03)	0.25	0.26	−1.95	2.46
	CONSTIPATION	6/23 (26.09%)	6/756 (0.79%)	32.87 (11.47–94.19)	44.12 (12.90–150.86)	94.15	2.32	0.86	3.79
	DIARRHOEA	1/23 (4.35%)	6/756 (0.79%)	5.48 (0.69–43.68)	5.68 (0.66–49.22)	3.17	0.68	−1.67	3.03
	DYSPEPSIA	1/23 (4.35%)	32/756 (4.23%)	1.03 (0.15–7.20)	1.03 (0.13–7.87)	0	−0.03	−2.2	2.14
	HICCUP	0/23 (0.00%)	8/756 (1.06%)	1.86 (0.11–31.22)	1.87 (0.11–33.43)	0.25	−0.35	−3.44	2.74
	MOUTH DRY	1/23 (4.35%)	6/756 (0.79%)	5.48 (0.69–43.68)	5.68 (0.66–49.22)	3.17	0.68	−1.67	3.03
	NAUSEA	3/23 (13.04%)	104/756 (13.76%)	0.95 (0.33–2.76)	0.94 (0.27–3.22)	0.01	−0.11	−1.68	1.46
	VOMITING	0/23 (0.00%)	44/756 (5.82%)	0.35 (0.02–5.59)	0.34 (0.02–5.70)	1.42	−1.25	−4.22	1.72
	PALPITATION	0/23 (0.00%)	17/756 (2.25%)	0.90 (0.06–14.55)	0.90 (0.05–15.40)	0.53	−0.63	−3.65	2.38
	HYPERGLYCAEMIA	0/23 (0.00%)	11/756 (1.46%)	1.37 (0.08–22.60)	1.38 (0.08–24.11)	0.34	−0.45	−3.51	2.6
	ANOREXIA	4/23 (17.39%)	7/756 (0.93%)	18.78 (5.91–59.70)	22.53 (6.08–83.50)	43.47	1.87	0.23	3.51
	APATHY	0/23 (0.00%)	5/756 (0.66%)	2.87 (0.16–50.39)	2.91 (0.16–54.12)	0.15	−0.24	−3.41	2.92
	DELIRIUM	3/23 (13.04%)	0/756 (0.00%)	220.79 (11.73–4156.65)	258.32 (12.92–5165.36)	98.99	1.83	−0.28	3.95
	DEPRESSION	2/23 (8.70%)	5/756 (0.66%)	13.15 (2.69–64.25)	14.30 (2.62–78.01)	16.18	1.27	−0.77	3.3
	DREAMING ABNORMAL	0/23 (0.00%)	17/756 (2.25%)	0.90 (0.06–14.55)	0.90 (0.05–15.40)	0.53	−0.63	−3.65	2.38
	INSOMNIA	8/23 (34.78%)	49/756 (6.48%)	5.37 (2.88–9.99)	7.70 (3.11–19.03)	26.36	1.7	0.52	2.87
	SLEEP DISORDER	0/23 (0.00%)	10/756 (1.32%)	1.50 (0.09–24.89)	1.51 (0.09–26.59)	0.31	−0.42	−3.49	2.65
	SOMNOLENCE	1/23 (4.35%)	8/756 (1.06%)	4.11 (0.54–31.51)	4.25 (0.51–35.47)	2.12	0.61	−1.69	2.92
	DYSPNOEA	0/23 (0.00%)	8/756 (1.06%)	1.86 (0.11–31.22)	1.87 (0.11–33.43)	0.25	−0.35	−3.44	2.74
	PHARYNGITIS	0/23 (0.00%)	14/756 (1.85%)	1.09 (0.07–17.70)	1.09 (0.06–18.82)	0.43	−0.55	−3.58	2.49
	DRUG QUALITY PROBLEM	1/23 (4.35%)	12/756 (1.59%)	2.74 (0.37–20.19)	2.82 (0.35–22.64)	1.04	0.48	−1.77	2.74
	PRURITUS	0/23 (0.00%)	51/756 (6.75%)	0.31 (0.02–4.82)	0.29 (0.02–4.87)	1.66	−1.38	−4.34	1.59
	RASH	0/23 (0.00%)	48/756 (6.35%)	0.33 (0.02–5.12)	0.31 (0.02–5.19)	1.56	−1.32	−4.29	1.64
	URTICARIA	1/23 (4.35%)	20/756 (2.65%)	1.64 (0.23–11.73)	1.67 (0.21–13.03)	0.25	0.26	−1.95	2.46
NRT	APPLICATION SITE REACTION	106/606 (17.49%)	0/173 (0.00%)	61.06 (3.81–977.56)	73.84 (4.56–1194.62)	35.03	0.35	−0.02	0.72
	CHEST PAIN	20/606 (3.30%)	4/173 (2.31%)	1.43 (0.49–4.12)	1.44 (0.49–4.28)	0.44	0.04	−0.8	0.89
	MALAISE	10/606 (1.65%)	20/173 (11.56%)	0.14 (0.07–0.30)	0.13 (0.06–0.28)	35.7	−1.19	−2.19	−0.18
	HYPERTENSION	10/606 (1.65%)	2/173 (1.16%)	1.43 (0.32–6.45)	1.43 (0.31–6.61)	0.22	−0.01	−1.18	1.16
	DIZZINESS	59/606 (9.74%)	13/173 (7.51%)	1.30 (0.73–2.31)	1.33 (0.71–2.48)	0.79	0.06	−0.43	0.54
	HEADACHE	34/606 (5.61%)	13/173 (7.51%)	0.75 (0.40–1.38)	0.73 (0.38–1.42)	0.86	−0.13	−0.75	0.5
	ABDOMINAL PAIN	17/606 (2.81%)	4/173 (2.31%)	1.21 (0.41–3.56)	1.22 (0.40–3.67)	0.12	0	−0.91	0.9
	CONSTIPATION	4/606 (0.66%)	8/173 (4.62%)	0.14 (0.04–0.47)	0.14 (0.04–0.46)	13.94	−1.15	−2.66	0.36
	DIARRHOEA	3/606 (0.50%)	4/173 (2.31%)	0.21 (0.05–0.95)	0.21 (0.05–0.95)	4.99	−0.85	−2.61	0.91
	DYSPEPSIA	14/606 (2.31%)	19/173 (10.98%)	0.21 (0.11–0.41)	0.19 (0.09–0.39)	24.95	−0.87	−1.75	0.02
	HICCUP	8/606 (1.32%)	0/173 (0.00%)	4.87 (0.28–84.01)	4.93 (0.28–85.81)	2.31	0.17	−1.18	1.53
	MOUTH DRY	3/606 (0.50%)	4/173 (2.31%)	0.21 (0.05–0.95)	0.21 (0.05–0.95)	4.99	−0.85	−2.61	0.91
	NAUSEA	56/606 (9.24%)	51/173 (29.48%)	0.31 (0.22–0.44)	0.24 (0.16–0.37)	46.52	−0.57	−1.02	−0.12
	VOMITING	32/606 (5.28%)	12/173 (6.94%)	0.76 (0.40–1.45)	0.75 (0.38–1.49)	0.69	−0.12	−0.77	0.53
	PALPITATION	16/606 (2.64%)	1/173 (0.58%)	4.57 (0.61–34.20)	4.66 (0.61–35.42)	2.68	0.19	−0.78	1.15
	HYPERGLYCAEMIA	11/606 (1.82%)	0/173 (0.00%)	6.59 (0.39–111.33)	6.70 (0.39–114.30)	3.19	0.22	−0.95	1.39
	ANOREXIA	1/606 (0.17%)	10/173 (5.78%)	0.03 (0.00–0.22)	0.03 (0.00–0.21)	30.48	−2.36	−4.56	−0.17
	APATHY	1/606 (0.17%)	4/173 (2.31%)	0.07 (0.01–0.63)	0.07 (0.01–0.63)	9.73	−1.5	−3.85	0.85
	DELIRIUM	0/606 (0.00%)	3/173 (1.73%)	0.04 (0.00–0.79)	0.04 (0.00–0.78)	10.55	−2.04	−5.26	1.19
	DEPRESSION	2/606 (0.33%)	5/173 (2.89%)	0.11 (0.02–0.58)	0.11 (0.02–0.58)	9.91	−1.26	−3.21	0.68
	DREAMING ABNORMAL	0/606 (0.00%)	17/173 (9.83%)	0.01 (0.00–0.14)	0.01 (0.00–0.12)	60.88	−3.9	−6.86	−0.94
	INSOMNIA	25/606 (4.13%)	32/173 (18.50%)	0.22 (0.14–0.37)	0.19 (0.11–0.33)	40.99	−0.82	−1.49	−0.15
	SLEEP DISORDER	4/606 (0.66%)	6/173 (3.47%)	0.19 (0.05–0.67)	0.18 (0.05–0.66)	8.37	−0.93	−2.48	0.62
	SOMNOLENCE	4/606 (0.66%)	5/173 (2.89%)	0.23 (0.06–0.84)	0.22 (0.06–0.84)	5.86	−0.81	−2.38	0.77
	DYSPNOEA	8/606 (1.32%)	0/173 (0.00%)	4.87 (0.28–84.01)	4.93 (0.28–85.81)	2.31	0.17	−1.18	1.53
	PHARYNGITIS	14/606 (2.31%)	0/173 (0.00%)	8.31 (0.50–138.66)	8.49 (0.50–143.09)	4.07	0.25	−0.8	1.29
	DRUG QUALITY PROBLEM	12/606 (1.98%)	1/173 (0.58%)	3.43 (0.45–26.16)	3.47 (0.45–26.91)	1.61	0.13	−0.97	1.24
	PRURITUS	48/606 (7.92%)	3/173 (1.73%)	4.57 (1.44–14.49)	4.87 (1.50–15.85)	8.42	0.25	−0.31	0.8
	RASH	47/606 (7.76%)	1/173 (0.58%)	13.42 (1.86–96.55)	14.46 (1.98–105.59)	11.99	0.3	−0.27	0.87
	URTICARIA	19/606 (3.14%)	2/173 (1.16%)	2.71 (0.64–11.53)	2.77 (0.64–12.00)	2.01	0.15	−0.73	1.03
Varenicline	APPLICATION SITE REACTION	0/150 (0.00%)	106/629 (16.85%)	0.02 (0.00–0.31)	0.02 (0.00–0.26)	29.26	−4.44	−7.34	−1.53
	CHEST PAIN	4/150 (2.67%)	20/629 (3.18%)	0.84 (0.29–2.42)	0.83 (0.28–2.48)	0.11	−0.22	−1.64	1.2
	MALAISE	17/150 (11.33%)	13/629 (2.07%)	5.48 (2.72–11.04)	6.06 (2.87–12.77)	28.09	1.37	0.5	2.24
	HYPERTENSION	2/150 (1.33%)	10/629 (1.59%)	0.84 (0.19–3.79)	0.84 (0.18–3.86)	0.05	−0.23	−2.08	1.63
	DIZZINESS	12/150 (8.00%)	60/629 (9.54%)	0.84 (0.46–1.52)	0.82 (0.43–1.58)	0.34	−0.21	−1.1	0.67
	HEADACHE	10/150 (6.67%)	37/629 (5.88%)	1.13 (0.58–2.23)	1.14 (0.55–2.35)	0.13	0.1	−0.88	1.08
	ABDOMINAL PAIN	3/150 (2.00%)	18/629 (2.86%)	0.70 (0.21–2.34)	0.69 (0.20–2.38)	0.34	−0.39	−1.97	1.19
	CONSTIPATION	2/150 (1.33%)	10/629 (1.59%)	0.84 (0.19–3.79)	0.84 (0.18–3.86)	0.05	−0.23	−2.08	1.63
	DIARRHOEA	3/150 (2.00%)	4/629 (0.64%)	3.14 (0.71–13.90)	3.19 (0.71–14.40)	2.53	0.65	−1.12	2.43
	DYSPEPSIA	18/150 (12.00%)	15/629 (2.38%)	5.03 (2.60–9.75)	5.58 (2.74–11.36)	27.6	1.33	0.49	2.17
	HICCUP	0/150 (0.00%)	8/629 (1.27%)	0.25 (0.01–4.23)	0.24 (0.01–4.23)	1.93	−1.45	−4.5	1.59
	MOUTH DRY	3/150 (2.00%)	4/629 (0.64%)	3.14 (0.71–13.90)	3.19 (0.71–14.40)	2.53	0.65	−1.12	2.43
	NAUSEA	48/150 (32.00%)	59/629 (9.38%)	3.41 (2.44–4.78)	4.55 (2.94–7.03)	52.3	1.17	0.65	1.69
	VOMITING	12/150 (8.00%)	32/629 (5.09%)	1.57 (0.83–2.98)	1.62 (0.81–3.23)	1.93	0.43	−0.5	1.35
	PALPITATION	1/150 (0.67%)	16/629 (2.54%)	0.26 (0.04–1.96)	0.26 (0.03–1.95)	2	−1.16	−3.32	0.99
	HYPERGLYCAEMIA	0/150 (0.00%)	11/629 (1.75%)	0.18 (0.01–3.06)	0.18 (0.01–3.05)	2.66	−1.73	−4.73	1.28
	ANOREXIA	6/150 (4.00%)	5/629 (0.79%)	5.03 (1.56–16.27)	5.20 (1.57–17.27)	8.94	1.08	−0.3	2.46
	APATHY	4/150 (2.67%)	1/629 (0.16%)	16.77 (1.89–148.99)	17.21 (1.91–155.08)	11.94	1.21	−0.54	2.97
	DELIRIUM	0/150 (0.00%)	3/629 (0.48%)	0.60 (0.03–11.48)	0.59 (0.03–11.58)	0.72	−0.82	−4.05	2.4
	DEPRESSION	3/150 (2.00%)	4/629 (0.64%)	3.14 (0.71–13.90)	3.19 (0.71–14.40)	2.53	0.65	−1.12	2.43
	DREAMING ABNORMAL	17/150 (11.33%)	0/629 (0.00%)	146.03 (8.83–2414.75)	165.04 (9.86–2761.55)	72.88	2.01	1.03	2.98
	INSOMNIA	24/150 (16.00%)	33/629 (5.25%)	3.05 (1.86–5.00)	3.44 (1.97–6.02)	20.65	1.04	0.33	1.74
	SLEEP DISORDER	6/150 (4.00%)	4/629 (0.64%)	6.29 (1.80–22.01)	6.51 (1.81–23.37)	10.82	1.17	−0.24	2.57
	SOMNOLENCE	4/150 (2.67%)	5/629 (0.79%)	3.35 (0.91–12.34)	3.42 (0.91–12.89)	3.72	0.77	−0.82	2.36
	DYSPNOEA	0/150 (0.00%)	8/629 (1.27%)	0.25 (0.01–4.23)	0.24 (0.01–4.23)	1.93	−1.45	−4.5	1.59
	PHARYNGITIS	0/150 (0.00%)	14/629 (2.23%)	0.14 (0.01–2.40)	0.14 (0.01–2.38)	3.4	−1.96	−4.94	1.02
	DRUG QUALITY PROBLEM	0/150 (0.00%)	13/629 (2.07%)	0.15 (0.01–2.59)	0.15 (0.01–2.57)	3.15	−1.89	−4.88	1.1
	PRURITUS	3/150 (2.00%)	48/629 (7.63%)	0.26 (0.08–0.83)	0.25 (0.08–0.80)	6.28	−1.46	−2.97	0.04
	RASH	1/150 (0.67%)	47/629 (7.47%)	0.09 (0.01–0.64)	0.08 (0.01–0.61)	9.7	−2.39	−4.47	−0.3
	URTICARIA	1/150 (0.67%)	20/629 (3.18%)	0.21 (0.03–1.55)	0.20 (0.03–1.53)	2.92	−1.39	−3.53	0.75

**Table 8:** SOC-level disproportionality analysis after excluding ICSRs with age < 18 years (*n* = 763 ICSRs).

Medication	WHO-ART SOC	Target ICSR, *n*/*N* (%)	Comparator ICSR, *n*/*N* (%)	PRR (95% CI)	ROR (95% CI)	IC	IC025	IC975	χ^2^
Bupropion	Body as a whole—general disorders	5/23 (21.74%)	121/740 (16.35%)	1.33 (0.60–2.94)	1.42 (0.52–3.90)	0.27	−1.06	1.6	0.47
	Central & peripheral nervous system disorders	5/23 (21.74%)	125/740 (16.89%)	1.29 (0.58–2.84)	1.37 (0.50–3.75)	0.23	−1.09	1.56	0.37
	Gastro-intestinal system disorders	10/23 (43.48%)	203/740 (27.43%)	1.58 (0.98–2.56)	2.03 (0.88–4.71)	0.52	−0.54	1.57	2.85
	Psychiatric disorders	13/23 (56.52%)	125/740 (16.89%)	3.35 (2.26–4.95)	6.40 (2.74–14.91)	1.39	0.4	2.37	23.65
	Respiratory system disorders	3/23 (13.04%)	46/740 (6.22%)	2.10 (0.70–6.25)	2.26 (0.65–7.90)	0.64	−0.96	2.24	1.73
	Skin and appendages disorders	5/23 (21.74%)	136/740 (18.38%)	1.18 (0.54–2.61)	1.23 (0.45–3.38)	0.14	−1.19	1.47	0.17
NRT	Application site disorders	107/590 (18.14%)	0/173 (0.00%)	63.30 (3.95–1013.36)	77.15 (4.77–1248.22)	0.36	−0.01	0.73	36.49
	Body as a whole—general disorders	95/590 (16.10%)	31/173 (17.92%)	0.90 (0.62–1.30)	0.88 (0.56–1.37)	−0.04	−0.41	0.32	0.32
	Cardiovascular disorders, general	13/590 (2.20%)	5/173 (2.89%)	0.76 (0.28–2.11)	0.76 (0.27–2.15)	−0.16	−1.17	0.85	0.27
	Central & peripheral nervous system disorders	99/590 (16.78%)	31/173 (17.92%)	0.94 (0.65–1.35)	0.92 (0.59–1.44)	−0.03	−0.39	0.33	0.12
	Gastro-intestinal system disorders	127/590 (21.53%)	86/173 (49.71%)	0.43 (0.35–0.54)	0.28 (0.19–0.40)	−0.37	−0.67	−0.08	52.81
	Hearing and vestibular disorders	7/590 (1.19%)	2/173 (1.16%)	1.03 (0.22–4.90)	1.03 (0.21–4.99)	−0.12	−1.48	1.24	0
	Heart rate and rhythm disorders	18/590 (3.05%)	2/173 (1.16%)	2.64 (0.62–11.26)	2.69 (0.62–11.71)	0.15	−0.76	1.05	1.88
	Liver and biliary system disorders	3/590 (0.51%)	0/173 (0.00%)	2.06 (0.11–39.71)	2.07 (0.11–40.22)	−0.03	−2.06	2.01	0.88
	Metabolic and nutritional disorders	22/590 (3.73%)	4/173 (2.31%)	1.61 (0.56–4.62)	1.64 (0.56–4.81)	0.08	−0.73	0.88	0.82
	Musculo-skeletal system disorders	19/590 (3.22%)	3/173 (1.73%)	1.86 (0.56–6.20)	1.89 (0.55–6.45)	0.1	−0.78	0.97	1.06
	Platelet, bleeding & clotting disorders	4/590 (0.68%)	0/173 (0.00%)	2.65 (0.14–48.98)	2.66 (0.14–49.70)	0.04	−1.78	1.86	1.18
	Psychiatric disorders	69/590 (11.69%)	69/173 (39.88%)	0.29 (0.22–0.39)	0.20 (0.13–0.30)	−0.63	−1.03	−0.23	71.75
	Red blood cell disorders	3/590 (0.51%)	0/173 (0.00%)	2.06 (0.11–39.71)	2.07 (0.11–40.22)	−0.03	−2.06	2.01	0.88
	Reproductive disorders, female	3/590 (0.51%)	0/173 (0.00%)	2.06 (0.11–39.71)	2.07 (0.11–40.22)	−0.03	−2.06	2.01	0.88
	Respiratory system disorders	41/590 (6.95%)	8/173 (4.62%)	1.50 (0.72–3.14)	1.54 (0.71–3.35)	0.09	−0.5	0.68	1.2
	Secondary terms—events	66/590 (11.19%)	1/173 (0.58%)	19.35 (2.71–138.43)	21.66 (2.98–157.27)	0.33	−0.15	0.8	18.79
	Skin and appendages disorders	129/590 (21.86%)	12/173 (6.94%)	3.15 (1.79–5.56)	3.75 (2.02–6.97)	0.24	−0.09	0.56	19.79
	Special senses other, disorders	6/590 (1.02%)	0/173 (0.00%)	3.83 (0.22–67.61)	3.86 (0.22–68.84)	0.13	−1.41	1.67	1.77
	Urinary system disorders	4/590 (0.68%)	4/173 (2.31%)	0.29 (0.07–1.16)	0.29 (0.07–1.17)	−0.67	−2.27	0.94	3.44
	Vascular (extracardiac) disorders	11/590 (1.86%)	0/173 (0.00%)	6.77 (0.40–114.34)	6.89 (0.40–117.46)	0.23	−0.94	1.4	3.27
	Vision disorders	11/590 (1.86%)	0/173 (0.00%)	6.77 (0.40–114.34)	6.89 (0.40–117.46)	0.23	−0.94	1.4	3.27
	White cell and RES disorders	6/590 (1.02%)	0/173 (0.00%)	3.83 (0.22–67.61)	3.86 (0.22–68.84)	0.13	−1.41	1.67	1.77
Varenicline	Body as a whole—general disorders	26/150 (17.33%)	100/613 (16.31%)	1.06 (0.72–1.57)	1.08 (0.67–1.73)	0.05	−0.58	0.68	0.09
	Cardiovascular disorders, general	5/150 (3.33%)	13/613 (2.12%)	1.57 (0.57–4.34)	1.59 (0.56–4.54)	0.34	−1.02	1.7	0.77
	Central & peripheral nervous system disorders	26/150 (17.33%)	104/613 (16.97%)	1.02 (0.69–1.51)	1.03 (0.64–1.65)	0.01	−0.62	0.64	0.01
	Gastro-intestinal system disorders	76/150 (50.67%)	137/613 (22.35%)	2.27 (1.83–2.81)	3.57 (2.46–5.18)	0.84	0.42	1.25	48.02
	Metabolic and nutritional disorders	3/150 (2.00%)	23/613 (3.75%)	0.53 (0.16–1.75)	0.52 (0.16–1.77)	−0.66	−2.21	0.89	1.12
	Psychiatric disorders	56/150 (37.33%)	82/613 (13.38%)	2.79 (2.09–3.73)	3.86 (2.57–5.78)	1.01	0.53	1.48	46.68
	Respiratory system disorders	5/150 (3.33%)	44/613 (7.18%)	0.46 (0.19–1.15)	0.45 (0.17–1.14)	−0.85	−2.11	0.4	2.96
	Skin and appendages disorders	7/150 (4.67%)	134/613 (21.86%)	0.21 (0.10–0.45)	0.17 (0.08–0.38)	−1.86	−2.91	−0.8	23.65
	Urinary system disorders	4/150 (2.67%)	4/613 (0.65%)	4.09 (1.03–16.15)	4.17 (1.03–16.88)	0.85	−0.77	2.47	4.71

Abbreviation: RES: reticuloendothelial system.

**Table 9:** PT-level disproportionality analysis after excluding ICSRs with age < 18 years (*n* = 763 ICSRs).

Medication	Preferred Term (PT)	Target ICSR, *n*/*N* (%)	Comparator ICSR, *n*/*N* (%)	PRR (95% CI)	ROR (95% CI)	χ^2^	IC	IC025	IC975
Bupropion	MALAISE	3/23 (13.04%)	27/740 (3.65%)	3.57 (1.17–10.94)	3.96 (1.11–14.15)	5.21	1.02	−0.61	2.66
	HEADACHE	3/23 (13.04%)	44/740 (5.95%)	2.19 (0.74–6.55)	2.37 (0.68–8.29)	1.94	0.68	−0.93	2.28
	CONSTIPATION	6/23 (26.09%)	6/740 (0.81%)	32.17 (11.23–92.20)	43.18 (12.63–147.65)	92.06	2.31	0.85	3.78
	NAUSEA	3/23 (13.04%)	102/740 (13.78%)	0.95 (0.32–2.76)	0.94 (0.27–3.21)	0.01	−0.11	−1.68	1.46
	ANOREXIA	4/23 (17.39%)	7/740 (0.95%)	18.39 (5.78–58.43)	22.05 (5.95–81.72)	42.46	1.86	0.23	3.5
	DELIRIUM	3/23 (13.04%)	0/740 (0.00%)	216.13 (11.48–4068.71)	252.85 (12.64–5056.20)	96.9	1.83	−0.29	3.95
	INSOMNIA	8/23 (34.78%)	49/740 (6.62%)	5.25 (2.82–9.78)	7.52 (3.04–18.60)	25.59	1.68	0.5	2.85
	COUGHING	3/23 (13.04%)	7/740 (0.95%)	13.79 (3.81–49.96)	15.71 (3.78–65.22)	25.24	1.57	−0.2	3.35
	DERMATITIS CONTACT	3/23 (13.04%)	2/740 (0.27%)	48.26 (8.47–275.08)	55.35 (8.76–349.75)	55.9	1.75	−0.19	3.7
NRT	APPLICATION SITE OEDEMA	11/590 (1.86%)	0/173 (0.00%)	6.77 (0.40–114.34)	6.89 (0.40–117.46)	3.27	0.23	−0.94	1.4
	APPLICATION SITE REACTION	102/590 (17.29%)	0/173 (0.00%)	60.36 (3.77–966.53)	72.81 (4.50–1178.33)	34.52	0.36	−0.02	0.74
	ALLERGIC REACTION	3/590 (0.51%)	0/173 (0.00%)	2.06 (0.11–39.71)	2.07 (0.11–40.22)	0.88	−0.03	−2.06	2.01
	ASTHENIA	3/590 (0.51%)	1/173 (0.58%)	0.88 (0.09–8.40)	0.88 (0.09–8.51)	0.01	−0.28	−2.21	1.65
	CHEST PAIN	19/590 (3.22%)	4/173 (2.31%)	1.39 (0.48–4.04)	1.41 (0.47–4.19)	0.38	0.04	−0.83	0.9
	FEVER	3/590 (0.51%)	0/173 (0.00%)	2.06 (0.11–39.71)	2.07 (0.11–40.22)	0.88	−0.03	−2.06	2.01
	LEG PAIN	5/590 (0.85%)	0/173 (0.00%)	3.24 (0.18–58.28)	3.26 (0.18–59.24)	1.48	0.09	−1.57	1.75
	MALAISE	10/590 (1.69%)	20/173 (11.56%)	0.15 (0.07–0.31)	0.13 (0.06–0.29)	34.47	−1.18	−2.18	−0.17
	MEDICINE INEFFECTIVE	21/590 (3.56%)	0/173 (0.00%)	12.66 (0.77–207.94)	13.10 (0.79–217.38)	6.33	0.29	−0.57	1.15
	OEDEMA	9/590 (1.53%)	0/173 (0.00%)	5.59 (0.33–95.63)	5.67 (0.33–97.90)	2.67	0.2	−1.08	1.48
	PAIN	4/590 (0.68%)	0/173 (0.00%)	2.65 (0.14–48.98)	2.66 (0.14–49.70)	1.18	0.04	−1.78	1.86
	TEMPERATURE CHANGED SENSATION	5/590 (0.85%)	0/173 (0.00%)	3.24 (0.18–58.28)	3.26 (0.18–59.24)	1.48	0.09	−1.57	1.75
	WITHDRAWAL SYNDROME	4/590 (0.68%)	0/173 (0.00%)	2.65 (0.14–48.98)	2.66 (0.14–49.70)	1.18	0.04	−1.78	1.86
	HYPERTENSION	9/590 (1.53%)	2/173 (1.16%)	1.32 (0.29–6.05)	1.32 (0.28–6.19)	0.13	−0.04	−1.26	1.19
	CONVULSIONS GRAND MAL	3/590 (0.51%)	0/173 (0.00%)	2.06 (0.11–39.71)	2.07 (0.11–40.22)	0.88	−0.03	−2.06	2.01
	DIZZINESS	57/590 (9.66%)	13/173 (7.51%)	1.29 (0.72–2.29)	1.32 (0.70–2.47)	0.74	0.06	−0.43	0.55
	HEADACHE	34/590 (5.76%)	13/173 (7.51%)	0.77 (0.41–1.42)	0.75 (0.39–1.46)	0.71	−0.12	−0.74	0.51
	VERTIGO	4/590 (0.68%)	0/173 (0.00%)	2.65 (0.14–48.98)	2.66 (0.14–49.70)	1.18	0.04	−1.78	1.86
	ABDOMINAL PAIN	17/590 (2.88%)	4/173 (2.31%)	1.25 (0.42–3.65)	1.25 (0.42–3.78)	0.16	0	−0.9	0.91
	CONSTIPATION	4/590 (0.68%)	8/173 (4.62%)	0.15 (0.04–0.48)	0.14 (0.04–0.47)	13.46	−1.14	−2.65	0.37
	DIARRHOEA	3/590 (0.51%)	4/173 (2.31%)	0.22 (0.05–0.97)	0.22 (0.05–0.97)	4.79	−0.84	−2.6	0.92
	DYSPEPSIA	13/590 (2.20%)	19/173 (10.98%)	0.20 (0.10–0.40)	0.18 (0.09–0.38)	25.66	−0.92	−1.83	−0.01
	GLOSSITIS	4/590 (0.68%)	0/173 (0.00%)	2.65 (0.14–48.98)	2.66 (0.14–49.70)	1.18	0.04	−1.78	1.86
	GUM HYPERPLASIA	3/590 (0.51%)	0/173 (0.00%)	2.06 (0.11–39.71)	2.07 (0.11–40.22)	0.88	−0.03	−2.06	2.01
	HICCUP	8/590 (1.36%)	0/173 (0.00%)	5.01 (0.29–86.29)	5.06 (0.29–88.17)	2.37	0.18	−1.17	1.53
	MOUTH DRY	3/590 (0.51%)	4/173 (2.31%)	0.22 (0.05–0.97)	0.22 (0.05–0.97)	4.79	−0.84	−2.6	0.92
	NAUSEA	54/590 (9.15%)	51/173 (29.48%)	0.31 (0.22–0.44)	0.24 (0.16–0.37)	46.58	−0.59	−1.05	−0.13
	STOMATITIS	4/590 (0.68%)	0/173 (0.00%)	2.65 (0.14–48.98)	2.66 (0.14–49.70)	1.18	0.04	−1.78	1.86
	STOMATITIS ULCERATIVE	4/590 (0.68%)	0/173 (0.00%)	2.65 (0.14–48.98)	2.66 (0.14–49.70)	1.18	0.04	−1.78	1.86
	TOOTH DISORDER	5/590 (0.85%)	0/173 (0.00%)	3.24 (0.18–58.28)	3.26 (0.18–59.24)	1.48	0.09	−1.57	1.75
	VOMITING	31/590 (5.25%)	12/173 (6.94%)	0.76 (0.40–1.44)	0.74 (0.37–1.48)	0.71	−0.13	−0.78	0.53
	APPLICATION SITE PAIN	6/590 (1.02%)	0/173 (0.00%)	3.83 (0.22–67.61)	3.86 (0.22–68.84)	1.77	0.13	−1.41	1.67
	PALPITATION	15/590 (2.54%)	1/173 (0.58%)	4.40 (0.59–33.06)	4.49 (0.59–34.21)	2.51	0.18	−0.81	1.18
	TACHYCARDIA	3/590 (0.51%)	1/173 (0.58%)	0.88 (0.09–8.40)	0.88 (0.09–8.51)	0.01	−0.28	−2.21	1.65
	HEPATIC FUNCTION ABNORMAL	3/590 (0.51%)	0/173 (0.00%)	2.06 (0.11–39.71)	2.07 (0.11–40.22)	0.88	−0.03	−2.06	2.01
	DIABETES MELLITUS	3/590 (0.51%)	0/173 (0.00%)	2.06 (0.11–39.71)	2.07 (0.11–40.22)	0.88	−0.03	−2.06	2.01
	HYPERGLYCAEMIA	11/590 (1.86%)	0/173 (0.00%)	6.77 (0.40–114.34)	6.89 (0.40–117.46)	3.27	0.23	−0.94	1.4
	XEROPHTHALMIA	3/590 (0.51%)	1/173 (0.58%)	0.88 (0.09–8.40)	0.88 (0.09–8.51)	0.01	−0.28	−2.21	1.65
	ARTHRALGIA	3/590 (0.51%)	0/173 (0.00%)	2.06 (0.11–39.71)	2.07 (0.11–40.22)	0.88	−0.03	−2.06	2.01
	MYALGIA	11/590 (1.86%)	1/173 (0.58%)	3.23 (0.42–24.81)	3.27 (0.42–25.49)	1.43	0.12	−1.02	1.27
	SKELETAL PAIN	6/590 (1.02%)	1/173 (0.58%)	1.76 (0.21–14.51)	1.77 (0.21–14.78)	0.28	−0.03	−1.52	1.45
	AGGRESSIVE REACTION	5/590 (0.85%)	1/173 (0.58%)	1.47 (0.17–12.47)	1.47 (0.17–12.67)	0.12	−0.09	−1.69	1.51
	ANXIETY	9/590 (1.53%)	1/173 (0.58%)	2.64 (0.34–20.69)	2.66 (0.34–21.18)	0.93	0.08	−1.18	1.33
	DRUG DEPENDENCE	19/590 (3.22%)	0/173 (0.00%)	11.48 (0.70–189.21)	11.84 (0.71–197.11)	5.71	0.29	−0.62	1.19
	HALLUCINATION	4/590 (0.68%)	1/173 (0.58%)	1.17 (0.13–10.43)	1.17 (0.13–10.57)	0.02	−0.17	−1.91	1.57
	INSOMNIA	25/590 (4.24%)	32/173 (18.50%)	0.23 (0.14–0.38)	0.19 (0.11–0.34)	39.35	−0.81	−1.48	−0.15
	PARONIRIA	4/590 (0.68%)	3/173 (1.73%)	0.39 (0.09–1.73)	0.39 (0.09–1.75)	1.64	−0.52	−2.16	1.12
	SLEEP DISORDER	4/590 (0.68%)	6/173 (3.47%)	0.20 (0.06–0.68)	0.19 (0.05–0.68)	8.05	−0.92	−2.47	0.63
	SOMNOLENCE	4/590 (0.68%)	5/173 (2.89%)	0.23 (0.06–0.86)	0.23 (0.06–0.86)	5.62	−0.8	−2.37	0.77
	SUICIDE ATTEMPT	4/590 (0.68%)	0/173 (0.00%)	2.65 (0.14–48.98)	2.66 (0.14–49.70)	1.18	0.04	−1.78	1.86
	ANAEMIA	3/590 (0.51%)	0/173 (0.00%)	2.06 (0.11–39.71)	2.07 (0.11–40.22)	0.88	−0.03	−2.06	2.01
	COUGHING	4/590 (0.68%)	6/173 (3.47%)	0.20 (0.06–0.68)	0.19 (0.05–0.68)	8.05	−0.92	−2.47	0.63
	DYSPNOEA	8/590 (1.36%)	0/173 (0.00%)	5.01 (0.29–86.29)	5.06 (0.29–88.17)	2.37	0.18	−1.17	1.53
	LARYNGITIS	8/590 (1.36%)	0/173 (0.00%)	5.01 (0.29–86.29)	5.06 (0.29–88.17)	2.37	0.18	−1.17	1.53
	PHARYNGITIS	14/590 (2.37%)	0/173 (0.00%)	8.54 (0.51–142.41)	8.73 (0.52–147.06)	4.18	0.26	−0.79	1.3
	PNEUMONITIS	3/590 (0.51%)	0/173 (0.00%)	2.06 (0.11–39.71)	2.07 (0.11–40.22)	0.88	−0.03	−2.06	2.01
	DRUG ADMINISTRATION ERROR	6/590 (1.02%)	1/173 (0.58%)	1.76 (0.21–14.51)	1.77 (0.21–14.78)	0.28	−0.03	−1.52	1.45
	DRUG QUALITY PROBLEM	12/590 (2.03%)	1/173 (0.58%)	3.52 (0.46–26.87)	3.57 (0.46–27.66)	1.69	0.14	−0.96	1.24
	INAPPROPRIATE SCHEDULE OF DRUG ADMINISTRATION	8/590 (1.36%)	0/173 (0.00%)	5.01 (0.29–86.29)	5.06 (0.29–88.17)	2.37	0.18	−1.17	1.53
	INCORRECT DOSE ADMINISTERED	3/590 (0.51%)	0/173 (0.00%)	2.06 (0.11–39.71)	2.07 (0.11–40.22)	0.88	−0.03	−2.06	2.01
	INCORRECT DRUG ADMINISTRATION DURATION	11/590 (1.86%)	0/173 (0.00%)	6.77 (0.40–114.34)	6.89 (0.40–117.46)	3.27	0.23	−0.94	1.4
	INCORRECT TECHNIQUE IN DRUG USAGE PROCESS	20/590 (3.39%)	0/173 (0.00%)	12.07 (0.73–198.58)	12.47 (0.75–207.23)	6.02	0.29	−0.59	1.17
	MEDICATION ERROR	9/590 (1.53%)	1/173 (0.58%)	2.64 (0.34–20.69)	2.66 (0.34–21.18)	0.93	0.08	−1.18	1.33
	BULLOUS ERUPTION	13/590 (2.20%)	0/173 (0.00%)	7.95 (0.47–133.05)	8.11 (0.48–137.16)	3.88	0.25	−0.83	1.33
	LIVEDO RETICULARIS	3/590 (0.51%)	0/173 (0.00%)	2.06 (0.11–39.71)	2.07 (0.11–40.22)	0.88	−0.03	−2.06	2.01
	PRURITUS	43/590 (7.29%)	3/173 (1.73%)	4.20 (1.32–13.38)	4.45 (1.36–14.54)	7.28	0.24	−0.35	0.83
	RASH	42/590 (7.12%)	1/173 (0.58%)	12.32 (1.71–88.84)	13.18 (1.80–96.49)	10.76	0.3	−0.3	0.9
	RASH ERYTHEMATOUS	11/590 (1.86%)	0/173 (0.00%)	6.77 (0.40–114.34)	6.89 (0.40–117.46)	3.27	0.23	−0.94	1.4
	SKIN DISCOLOURATION	5/590 (0.85%)	0/173 (0.00%)	3.24 (0.18–58.28)	3.26 (0.18–59.24)	1.48	0.09	−1.57	1.75
	SKIN DISORDER	6/590 (1.02%)	0/173 (0.00%)	3.83 (0.22–67.61)	3.86 (0.22–68.84)	1.77	0.13	−1.41	1.67
	SKIN DRY	3/590 (0.51%)	0/173 (0.00%)	2.06 (0.11–39.71)	2.07 (0.11–40.22)	0.88	−0.03	−2.06	2.01
	SWEATING INCREASED	3/590 (0.51%)	2/173 (1.16%)	0.44 (0.07–2.61)	0.44 (0.07–2.64)	0.86	−0.49	−2.35	1.37
	URTICARIA	19/590 (3.22%)	2/173 (1.16%)	2.79 (0.66–11.84)	2.85 (0.66–12.34)	2.13	0.16	−0.72	1.04
	TASTE PERVERSION	5/590 (0.85%)	0/173 (0.00%)	3.24 (0.18–58.28)	3.26 (0.18–59.24)	1.48	0.09	−1.57	1.75
	CEREBRAL HAEMORRHAGE	5/590 (0.85%)	0/173 (0.00%)	3.24 (0.18–58.28)	3.26 (0.18–59.24)	1.48	0.09	−1.57	1.75
	FLUSHING	4/590 (0.68%)	0/173 (0.00%)	2.65 (0.14–48.98)	2.66 (0.14–49.70)	1.18	0.04	−1.78	1.86
	DIPLOPIA	5/590 (0.85%)	0/173 (0.00%)	3.24 (0.18–58.28)	3.26 (0.18–59.24)	1.48	0.09	−1.57	1.75
	VISION ABNORMAL	4/590 (0.68%)	0/173 (0.00%)	2.65 (0.14–48.98)	2.66 (0.14–49.70)	1.18	0.04	−1.78	1.86
	LYMPHADENOPATHY	6/590 (1.02%)	0/173 (0.00%)	3.83 (0.22–67.61)	3.86 (0.22–68.84)	1.77	0.13	−1.41	1.67
Varenicline	CHEST PAIN	4/150 (2.67%)	19/613 (3.10%)	0.86 (0.30–2.49)	0.86 (0.29–2.56)	0.08	−0.2	−1.62	1.23
	FATIGUE	3/150 (2.00%)	1/613 (0.16%)	12.26 (1.28–117.04)	12.49 (1.29–120.94)	7.8	1.01	−0.93	2.95
	MALAISE	17/150 (11.33%)	13/613 (2.12%)	5.34 (2.65–10.76)	5.90 (2.80–12.44)	27.08	1.34	0.47	2.21
	HYPOTENSION	3/150 (2.00%)	2/613 (0.33%)	6.13 (1.03–36.36)	6.23 (1.03–37.65)	5.19	0.87	−0.99	2.74
	DIZZINESS	12/150 (8.00%)	58/613 (9.46%)	0.85 (0.47–1.53)	0.83 (0.43–1.59)	0.31	−0.2	−1.09	0.68
	HEADACHE	10/150 (6.67%)	37/613 (6.04%)	1.10 (0.56–2.17)	1.11 (0.54–2.29)	0.08	0.07	−0.9	1.05
	ABDOMINAL PAIN	3/150 (2.00%)	18/613 (2.94%)	0.68 (0.20–2.28)	0.67 (0.20–2.32)	0.39	−0.41	−1.99	1.16
	DIARRHOEA	3/150 (2.00%)	4/613 (0.65%)	3.06 (0.69–13.55)	3.11 (0.69–14.03)	2.41	0.64	−1.14	2.41
	DYSPEPSIA	18/150 (12.00%)	14/613 (2.28%)	5.25 (2.67–10.32)	5.83 (2.83–12.03)	28.31	1.34	0.5	2.19
	MOUTH DRY	3/150 (2.00%)	4/613 (0.65%)	3.06 (0.69–13.55)	3.11 (0.69–14.03)	2.41	0.64	−1.14	2.41
	NAUSEA	48/150 (32.00%)	57/613 (9.30%)	3.44 (2.45–4.83)	4.59 (2.96–7.11)	52.33	1.16	0.64	1.68
	VOMITING	12/150 (8.00%)	31/613 (5.06%)	1.58 (0.83–3.01)	1.63 (0.82–3.26)	1.96	0.43	−0.5	1.35
	ANOREXIA	6/150 (4.00%)	5/613 (0.82%)	4.90 (1.52–15.85)	5.07 (1.53–16.83)	8.6	1.06	−0.32	2.44
	APATHY	4/150 (2.67%)	1/613 (0.16%)	16.35 (1.84–145.20)	16.77 (1.86–151.14)	11.6	1.2	−0.56	2.95
	DEPRESSION	3/150 (2.00%)	4/613 (0.65%)	3.06 (0.69–13.55)	3.11 (0.69–14.03)	2.41	0.64	−1.14	2.41
	DREAMING ABNORMAL	17/150 (11.33%)	0/613 (0.00%)	142.32 (8.61–2353.36)	160.84 (9.61–2691.43)	71.06	1.99	1.01	2.96
	INSOMNIA	24/150 (16.00%)	33/613 (5.38%)	2.97 (1.81–4.87)	3.35 (1.91–5.86)	19.65	1.01	0.3	1.72
	SLEEP DISORDER	6/150 (4.00%)	4/613 (0.65%)	6.13 (1.75–21.45)	6.34 (1.77–22.77)	10.44	1.14	−0.26	2.55
	SOMNOLENCE	4/150 (2.67%)	5/613 (0.82%)	3.27 (0.89–12.03)	3.33 (0.88–12.56)	3.54	0.75	−0.84	2.34
	COUGHING	3/150 (2.00%)	7/613 (1.14%)	1.75 (0.46–6.69)	1.77 (0.45–6.91)	0.69	0.34	−1.35	2.03
	SPUTUM INCREASED	3/150 (2.00%)	2/613 (0.33%)	6.13 (1.03–36.36)	6.23 (1.03–37.65)	5.19	0.87	−0.99	2.74
	PRURITUS	3/150 (2.00%)	43/613 (7.01%)	0.29 (0.09–0.91)	0.27 (0.08–0.88)	5.35	−1.36	−2.87	0.15

## Data Availability

The data presented in this study are available upon request from the corresponding author and KIDS owing to the inclusion of patient information and ethical concerns.

## Supplementary Materials

The following supporting information can be downloaded at: https://www.mdpi.com/article/10.3390/healthcare14203479/s1, Table S1. STROBE checklist (page numbers based on original submission). Table S2. READUS-PV checklist (page numbers based on original submission). Table S3. Distribution of WHO-UMC causality assessments and impact of causality-based restriction. Table S4. SOC-level disproportionality analysis of adverse event reporting among smoking-cessation medications (*n* = 279 ICSRs). Table S5. Disproportionality analysis of PT-Tobacco cessation medication (*n* = 279 ICSRs). Table S6. Sensitivity analysis of signals of disproportionate reporting using an alternative Gamma–Poisson shrinkage approach.

## Abbreviations

The following abbreviations are used in this manuscript:

ADE	Adverse Drug Event
AE	Adverse events
CI	Confidence Interval
IC	Information Component
NRT	Nicotine Replacement Therapy
OTC	Over-The-Counter
PRR	Proportional Reporting Ratio
ROR	Reporting Odds Ratio
SAE	Serious Adverse Events
