# Supplementary material for: Adverse Event Reporting Patterns of Tobacco Cessation Medications in Korea: A Disproportionality Analysis
Source: Healthcare (Basel). 2026 Oct 16;14(20):3479. doi: 10.3390/healthcare14203479 (PMC13704679; doi:10.3390/healthcare14203479)
Supplement: Supplementary file 1 [file healthcare-14-03479-s001.zip › healthcare-4496284-supplementary.pdf]

**Table S1.** STROBE checklist (based on original submission)

| | Item No. | Recommendation | Page No. | Relevant text from manuscript |
|---|---|---|---|---|
| **Title and abstract** | 1 | (*a*) Indicate the study's design with a commonly used term in the title or the abstract | 1 | Adverse Event Reporting Patterns of Tobacco Cessation Medications in Korea: A Disproportionality Analysis |
| | | (*b*) Provide in the abstract an informative and balanced summary of what was done and what was found | 1 | A total of 779 individual case safety reports (ICSRs) involving tobacco cessation medications were included. Nicotine replacement therapy (NRT) accounted for 606 ICSRs (77.79%), followed by varenicline (n=150, 19.26%) and bupropion (n=23, 2.95%). Age information was unavailable for 558 ICSRs (71.63%). Among ICSRs with documented age information, the 50–59-year age group was the most frequently represented. Fifty-two serious ICSRs (6.68%) were identified, comprising 49 ICSRs involving NRT and 3 involving bupropion. SOC-level disproportionality analyses identified signals of disproportionate reporting for psychiatric disorders for bupropion and psychiatric and gastrointestinal disorders for varenicline. All identified signals remained detectable after excluding reports involving individuals aged <18 years, whereas causality-based restriction altered the detected signal patterns. |
| **Introduction** | | | | |
| Background/rationale | 2 | Explain the scientific background and rationale for the investigation being reported | 2 | Pharmacovigilance databases provide valuable opportunities to identify rare or unexpected AEs that may not be detected in pre-marketing clinical trials [13]. Previous Korean pharmacovigilance research using Korea Adverse Event Reporting System database (KAERS DB) has primarily focused on signal detection of varenicline [17], while concurrent evaluation of AE reporting patterns across NRT, bupropion, and varenicline within the same national pharmacovigilance database remains limited. |

| | | | | |
|---|---|---|---|---|
| Objectives | 3 | State specific objectives, including any prespecified hypotheses | 2 | Therefore, the primary objective of this study was to characterize AE reporting patterns associated with NRT, bupropion, and varenicline in the KIDS KAERS DB and to identify potential signals of disproportionate reporting. Secondary objectives were to describe demographic characteristics, seriousness, temporal reporting patterns, and reporter characteristics at the individual case safety report (ICSR) level. |
| **Methods** | | | | |
| Study design | 4 | Present key elements of study design early in the paper | 2 | A disproportionality analysis using individual case safety reports from KIDS KAERS DB was conducted in accordance with the Strengthening the reporting of observational studies in epidemiology (STROBE) guidelines and the Reporting of A Disproportionality Analysis for DrUg Safety Signal Detection using Individual Case Safety Reports in Pharmacovigilance (READUS-PV) recommendations (Table S1 & S2) [18,19]. |
| Setting | 5 | Describe the setting, locations, and relevant dates, including periods of recruitment, exposure, follow-up, and data collection | 2-3 | This study analyzed adverse drug events (ADEs) related to tobacco cessation products, including bupropion, NRT, and varenicline spontaneously reported to the KIDS KAERS DB, constructed by the Korea Institute of Drug Safety & Risk Management (KIDS, Ministry of Food and Drug Safety), between January 1, 2014 and December 31, 2023. |
| Participants | 6 | (*a*) *Cohort study*—Give the eligibility criteria, and the sources and methods of selection of participants. Describe methods of follow-up<br>*Case-control study*—Give the eligibility criteria, and the sources and methods of case ascertainment and control selection. Give the rationale for the choice of cases and controls<br>*Cross-sectional study*—Give the eligibility criteria, and the sources and methods of selection of participants | 3 | To minimize potential indication bias, particularly for bupropion, only reports associated with tobacco cessation-related diagnoses or treatment indications were included. Reports with missing indication information or with indications that could not be clearly classified as tobacco cessation-related were excluded. Eligible cases were restricted to reports containing tobacco-related Korean Standard Classification Disease (KCD) codes, including nicotine dependence (F17), and tobacco use (Z72.0), or tobacco cessation- |

| | | | | |
|---|---|---|---|---|
| | | | | related efficacy terms, such as smoking cessation therapy, smoking cessation, tobacco use, tobacco withdrawal symptoms, tobacco dependence, or nicotine-related effects. The prespecified exclusion criteria were reports associated with non-tobacco cessation indications, including major depressive disorder, insomnia, or other psychiatric indications for which bupropion may be prescribed, and reports involving masked (MSK-coded) etiologic medications. |
| | | (*b*) *Cohort study*—For matched studies, give matching criteria and number of exposed and unexposed<br>*Case-control study*—For matched studies, give matching criteria and the number of controls per case | N/A | Not applicable: no matching of exposed/unexposed participants or cases/controls was used in this ICSR-based disproportionality analysis. |
| Variables | 7 | Clearly define all outcomes, exposures, predictors, potential confounders, and effect modifiers. Give diagnostic criteria, if applicable | 3-4 | The following data were extracted from KIDS KAERS DB: 1) patient demographics (sex and age), 2) medical histories and concurrent medications, and 3) ADE characteristics, including etiologic medication, occurrence date, causality assessment, ADE type, and seriousness. SAEs were defined as any ADEs involving congenital abnormalities or birth defects, life-threatening conditions, death, a persistent or significant disability or incapacity, hospitalization or prolonged existing hospitalizations, and other medically significant events, in accordance with the International Conference on Harmonization (ICH) E2D guidelines. A signal of disproportionate reporting (SDR) was considered present only when all of the following criteria were met: PRR $\geq$ 2, ROR $\geq$ 2, Pearson's chi-square $\geq$ 4, and IC025 > 0 [24]. |
| Data sources/ measurement | 8* | For each variable of interest, give sources of data and details of methods of assessment (measurement). Describe comparability of assessment methods if there is more than one group | 3 | ICSRs were identified using anonymized KAERS report number (KAERS_NO), which was used to link records belonging to the same safety report across the merged tables. AE terms in the KIDS KAERS DB were provided as MedDRA lowest level terms (LLTs), with World Health Organization-Adverse Reaction Terminology |

| | | | | |
|---|---|---|---|---|
| | | | | (WHO-ART) codes provided in the database. PT- and SOC-level analyses were based on the WHO-ART-coded variables using WHO-ART version 092. |
| Bias | 9 | Describe any efforts to address potential sources of bias | 3 | To minimize potential indication bias, particularly for bupropion, only reports associated with tobacco cessation-related diagnoses or treatment indications were included.<br>After merging the relevant KAERS DB tables, rows with identical values across all variables were considered exact duplicates and were removed. When multiple versions of the same ICSR were available, only the most recent version was retained for analysis in accordance with the KIDS KAERS DB data-use guidelines [21]. |
| Study size | 10 | Explain how the study size was arrived at | 5 | A total of 779 unique ICSRs involving tobacco cessation medications were included, comprising 1,356 unique ICSR-drug-PT combinations and 1,147 unique ICSR-drug-SOC combinations (Figure 1). |

Continued on next page

| | | | | |
|---|---|---|---|---|
| Quantitative variables | 11 | Explain how quantitative variables were handled in the analyses. If applicable, describe which groupings were chosen and why | 3 | Age was expressed as the median and interquartile range (IQR) based on the Kolmogorov-Smirnov normality test. |
| Statistical methods | 12 | (*a*) Describe all statistical methods, including those used to control for confounding | 3-4 | Descriptive statistics were performed to summarize the demographic and reporting characteristics of the included ICSRs and AE patterns. For each target medication, the comparator comprised ICSRs involving the other two smoking-cessation medications. Disproportionality analyses were performed for medication–SOC and medication-PT combinations reported in at least three unique ICSRs ($A \geq 3$)[22,23]. A signal of disproportionate reporting (SDR) was considered present only when all of the following criteria were met: $PRR \geq 2$, $ROR \geq 2$, Pearson's chi-square $\geq 4$, and $IC025 > 0$ [24]. When any cell of the $2 \times 2$ contingency table contained zero, 0.5 was added to all four cells using the Haldane–Anscombe correction for estimation of the PRR and ROR. Pearson's chi-square and the Bayesian Confidence Propagation Neural Network (BCPNN) were calculated using the original, uncorrected cell counts (Table 1). |
| | | (*b*) Describe any methods used to examine subgroups and interactions | 3-4 | Age- and sex-specific descriptive analyses were conducted using available information, and the proportion of missing demographic information was reported.<br>A sensitivity analysis was performed after excluding ICSRs with documented age <18 years while retaining those with missing age information. |
| | | (*c*) Explain how missing data were addressed | 3 | Missing demographic information was not imputed. Age- and sex-specific descriptive analyses were conducted using available information, and the proportion of missing demographic information was reported. |

| | | | | |
|---|---|---|---|---|
| | | (*d*) *Cohort study*—If applicable, explain how loss to follow-up was addressed<br>*Case-control study*—If applicable, explain how matching of cases and controls was addressed<br>*Cross-sectional study*—If applicable, describe analytical methods taking account of sampling strategy | N/A | ICSRs were selected using prespecified eligibility criteria; follow-up, case–control matching, and sampling-design adjustments were not applicable. |
| | | (*e*) Describe any sensitivity analyses | 4, 22, 33 | A sensitivity analysis was performed after excluding ICSRs with documented age <18 years while retaining those with missing age information. An additional sensitivity analysis was conducted by restricting drug–event relationships to those classified as "certain", "probable/likely", or "possible" according to WHO-UMC criteria to assess the influence of causality-based restriction on SDR detection. All other eligibility criteria and analytical procedures were maintained as in the primary analysis. As an additional methodological check, we also examined whether SDR classification changed when the Bayesian component was recalculated using an alternative Gamma–Poisson shrinkage approach (Table S6) [42]. |
| **Results** | | | | |
| Participants | 13* | (a) Report numbers of individuals at each stage of study—eg numbers potentially eligible, examined for eligibility, confirmed eligible, included in the study, completing follow-up, and analysed | 5 | Figure 1 |
| | | (b) Give reasons for non-participation at each stage | 3, 5 | Reports with missing indication information or with indications that could not be clearly classified as tobacco cessation-related were excluded. The prespecified exclusion criteria were reports associated with non-tobacco cessation indications, including major depressive disorder, insomnia, or other psychiatric indications for which bupropion may be prescribed, and reports involving masked (MSK-coded) etiologic medications. |

| | | | | |
|---|---|---|---|---|
| | | (c) Consider use of a flow diagram | 5 | Figure 1. Flow diagram of data preprocessing and ICSR selection. |
| Descriptive data | 14* | (a) Give characteristics of study participants (eg demographic, clinical, social) and information on exposures and potential confounders | 5 | Among ICSRs with available age information, the median age was 47 years (IQR 29), and the 50–59-year age group was the most frequently represented (n = 45, 20.36%). Sixteen of the 221 ICSRs with available age information (7.24%) involved individuals aged <18 years. Sex information was available for 571 ICSRs, of which 458 (80.21%) involved male individuals. Reports from the general public constituted the largest primary reporter group (n=341, 43.77%), followed by pharmacists (n=251, 32.22%). NRT accounted for 606 ICSRs (77.79%), followed by varenicline (n=150, 19.26%) and bupropion (n=23, 2.95%). Forty-seven ICSRs (6.03%) involved at least one concomitant medication. |
| | | (b) Indicate number of participants with missing data for each variable of interest | 5-6 | Age information was available for 221 ICSRs and was missing for 558 ICSRs (71.63%) (Table 2). Sex information was available for 571 ICSRs, of which 458 (80.21%) involved male individuals. |
| | | (c) *Cohort study*—Summarise follow-up time (eg, average and total amount) | N/A | N/A |
| Outcome data | 15* | *Cohort study*—Report numbers of outcome events or summary measures over time | N/A | N/A |
| | | *Case-control study*—Report numbers in each exposure category, or summary measures of exposure | N/A | N/A |
| | | *Cross-sectional study*—Report numbers of outcome events or summary measures | 9-10 | Among the 779 included ICSRs, 52 serious ICSRs were identified. The most frequently reported serious criteria were other medically important events (n=31, 59.62%) and hospitalization or prolonged hospitalization (n=22, 42.31%) (Table 4). As ICSR could meet more than one seriousness criterion, these categories were not mutually exclusive. |
| Main results | 16 | (*a*) Give unadjusted estimates and, if applicable, confounder-adjusted estimates and their precision (eg, | 10-20 | In the primary BCPNN analysis, SDRs were identified for skin and appendages disorders (PRR 6.43, ROR 8.54, $\chi^2$ 30.93, IC 0.90, IC025 |

| | | | |
|---|---|---|---|
| | 95% confidence interval). Make clear which confounders were adjusted for and why they were included | | 0.24) and application site disorders (PRR 50.37, ROR 59.46, $\chi^2$ 26.25, IC 1.08, IC025 0.15) with NRT, and for psychiatric disorders with varenicline (PRR 2.76, ROR 3.88, $\chi^2$ 21.83, IC 0.51, IC025 0.04). No SDRs were identified for bupropion (Table 5). |
| | (*b*) Report category boundaries when continuous variables were categorized | 6 | 0~9; 10~19; 20~29; 30~39; 40~49; 50~59; 60~69; 70~79; 80~89 for Age |
| | (*c*) If relevant, consider translating estimates of relative risk into absolute risk for a meaningful time period | 32 | As population-level exposure denominators were unavailable, the analyses cannot estimate AE incidence or absolute or relative risks. |

Continued on next page

| | | | | |
|---|---|---|---|---|
| Other analyses | 17 | Report other analyses done—eg analyses of subgroups and interactions, and sensitivity analyses | 22, 32 | In the sensitivity analysis excluding 16 ICSRs with documented age <18 years while retaining ICSRs with missing age information, 763 ICSRs were included (Table 8 & 9). In an additional sensitivity analysis restricted to drug–event relationships classified as certain, probable, or possible, 279 ICSRs were included. These findings indicate that the application of causality-based exclusion influenced the magnitude and classification of some disproportionality signals. |
| **Discussion** | | | | |
| Key results | 18 | Summarise key results with reference to study objectives | 29 | This nationwide pharmacovigilance study characterized adverse event reporting patterns for NRT, bupropion, and varenicline using 779 unique ICSRs from the KAERS DB. In the primary ICSR-level analysis, SDRs were identified for psychiatric disorders with both bupropion and varenicline and for gastrointestinal disorders with varenicline, whereas no SDR met the predefined criteria for NRT. At the PT level, SDRs included constipation, anorexia, and insomnia with bupropion, and malaise, dyspepsia, nausea, abnormal dreaming, and insomnia with varenicline. Although application site reaction, pruritus, and rash were frequently reported with NRT, none met all predefined SDR criteria. A total of 52 ICSRs (6.68%) were classified as serious, including 49 involving NRT and three involving bupropion, while none were reported for varenicline. |
| Limitations | 19 | Discuss limitations of the study, taking into account sources of potential bias or imprecision. Discuss both direction and magnitude of any potential bias | 32-33 | This study has several limitations that should be acknowledged. First, this study was based on a spontaneous reporting pharmacovigilance database, which is inherently subject to underreporting, reporting bias, and incomplete clinical information. Furthermore, causality-based restriction reduced the dataset from 779 to 279 ICSRs and altered the detected SDR patterns, implying the influence of report selection on signal detection. |

| | | | | |
|---|---|---|---|---|
| Interpretation | 20 | Give a cautious overall interpretation of results considering objectives, limitations, multiplicity of analyses, results from similar studies, and other relevant evidence | 29, 32-33 | Nevertheless, as this study focused on signal detection rather than comparative risk estimation, the observed differences should be interpreted as relative reporting patterns rather than evidence of differences in medication safety. |
| Generalisability | 21 | Discuss the generalisability (external validity) of the study results | 33 | Because the study was based on a single national pharmacovigilance system, the findings may also have limited generalizability to healthcare systems with different prescribing practices, tobacco control policies, and pharmacovigilance structures. |
| **Other information** | | | | |
| Funding | 22 | Give the source of funding and the role of the funders for the present study and, if applicable, for the original study on which the present article is based | 34 | Funding: This research was partially supported by National Research Foundation of Korea, funded by Ministry of Science and ICT (MSIT) (grant number RS-2026-25474310) and the Ministry of Food and Drug Safety (grant number 21153MFDS601). |

*Give information separately for cases and controls in case-control studies and, if applicable, for exposed and unexposed groups in cohort and cross-sectional studies.

**Note:** An Explanation and Elaboration article discusses each checklist item and gives methodological background and published examples of transparent reporting. The STROBE checklist is best used in conjunction with this article (freely available on the Web sites of PLoS Medicine at http://www.plosmedicine.org/, Annals of Internal Medicine at http://www.annals.org/, and Epidemiology at http://www.epidem.com/). Information on the STROBE Initiative is available at www.strobe-statement.org.

**Table S2.** READUS-PV checklist

| **Section and topic** | **Item #** | **Checklist item** | **Location where item is reported** |
|---|---|---|---|
| **Title** | | | |
| | *1a* | *If disproportionality analyses are a prominent component of the published study, the study should be identified as a "disproportionality analysis". The type of data and name of the database(s) should be specified.* | Page 1 |
| | *1b* | *Report the name of adverse event(s) and/or drug(s) under study, when applicable.* | Page 1 |
| **Introduction** | | | |
| Background | *2a* | *Describe the drug(s) and its utilization, the nature of the adverse event(s) under study and its frequency, and the existing knowledge on the drug-event combination.* | Page 2 |
| | *2b* | *Specify the rationale for performing the analysis, e.g., as part of routine pharmacovigilance, to investigate an overall safety profile, or to assess a pre-specified hypothesis.* | Page 2 |
| | *2c* | *Explain why ICSR databases and disproportionality analysis are suitable to fill the knowledge gap.* | Page 2 |
| Objectives | *3* | *State specific objectives, identifying the adverse event(s), the drug(s), and the reference group, including any pre-specified hypothesis, if applicable.* | Page 2, Page 4 |
| **Methods** | | | |
| Study design | *4a* | *Identify the study (i.e., "disproportionality analysis") and the type of data used (e.g., "individual case safety reports").* | Page 2 |
| | *4b* | *Provide an outline of the entire study design, including primary and sensitivity analyses performed, and other designs such as case-by-case analysis or literature review.* | Page 2–4, Page 22, Page 32 |
| Data description, access, and pre-processing | *5a* | *Specify the name of the database(s), the database(s) custodian, and the coverage. Specify the type/number of drugs included within the database and the thesaurus, taxonomies, or ontologies used for coding drugs and events.* | Page 2–3 |

| | | | |
|---|---|---|---|
| | *5b* | *Specify the extraction dates and describe and justify all choices used for data pre-processing, including any data transformation or exclusion, if appropriate.* | Page 2–3, Page 5, Figure 1 |
| Variables definition | *6a* | *Describe the study population, including any restriction.* | Page 2–3 |
| | *6b* | *Describe the nature and the meaning of key variables assessed in the work.* | Page 2–4 |
| | *6c* | *Specify and justify any grouping of drugs or events. For drugs, specify and justify whether active ingredients/trade names/salts were considered and/or the selected role.* | Page 2–3 |
| | *6d* | *Describe any additional data source used, the type of data, and how they interact with ICSRs.* | N/A |
| Statistical methods | *7a* | *Present any descriptive analysis performed, specifying variables investigated, statistical tests, and significance thresholds.* | Page 2–3 |
| | *7b* | *Describe the measure(s) selected for the disproportionality analysis including any threshold used to identify signals of disproportionate reporting. Explain the reason for this choice if applicable.* | Page 4–5, Table 1 |
| | *7c* | *Clearly describe any sensitivity analysis and any tool to control confounding, including any restriction, subgroup, stratification, adjustment, or interaction.* | Page 4, Page 21–28, Page 31 |
| | *7d* | *Specify the variables and methods used for the case-by-case analysis, including any algorithm or criteria used to assess causality, if performed.* | Page 3, Page 9–10 |
| | *7e* | *Specify any statistical methods used for other data sources.* | N/A |
| **Results** | | | |
| Participants | *8a* | *Specify the number of individual case safety reports included at each stage, including reasons for exclusion.* | Page 5, Figure 1, Page 21, Supplementary Table S3 |
| | *8b* | *Provide key demographic and clinical characteristics of cases, if possible comparing cases with any appropriate reference group.* | Page 5–9, Tables 2–3, Figure 2 |
| Disproportionality | *9* | *Present all results including confidence intervals. Present also results of sensitivity analyses, if performed.* | Page 10–28, Table 5–9, |

| | | | |
|---|---|---|---|
| analysis | | | Supplementary Table S3–S6 |
| Case-by-case analysis | *10* | *Present the case-by-case analysis of key variables. Present the causality assessment, if applicable.* | Page 6–7, Page 9–10, Table 2, Table 4 |
| **Discussion** | | | |
| Key results | *11* | *Discuss key results with reference to study objectives and contextualize them within the current literature and other consulted sources. Clearly discriminate between expected reactions and emerging safety signals.* | Page 29-32 |
| External validity | *12a* | *Discuss the external validity of the results to the general population.* | Page 33 |
| | *12b* | *Discuss the potential relevance of results in clinical practice* | Page 29, Page 31–32 |
| | *12c* | *Propose further study designs if applicable* | Page 32–33 |
| Limitations | *13* | *Present general limitations, making clear that disproportionality analysis alone cannot prove causation or measure incidence, and specific limitations, including confounding and reporting bias and efforts to mitigate them.* | Page 3–4, Page 32–33 |
| **Declarations** | | | |
| | *14a* | *Provide the source of funding/sponsorship and the role of the funders/sponsors for the present study and for any original study on which the present article is based.* | Page 34 |
| | *14b* | *Clearly identify potential commercial and intellectual conflicts of interest (e.g., link to any drug/event investigated, whether financial, legal action, or software used).* | Page 34 |
| | *14c* | *Declare any institutional approval needed or granted in the investigation.* | Page 3, Page 34 |
| | *14d* | *Include a statement on data availability, code availability (including the version of the statistical software used), and protocol registration.* | Page 3–4, Page 34 |

**Table S3.** Distribution of WHO-UMC causality assessments and impact of causality-based restriction

| Measure | Overall | Bupropion | NRT | Varenicline |
|---|---|---|---|---|
| **A. Causality record status among unique drug–event relationships, n (%)** | **N = 1,531** | **N = 88** | **N = 1,188** | **N = 255** |
| Certain | 30 (1.96) | 3 (3.41) | 23 (1.94) | 4 (1.57) |
| Probable/Likely | 140 (9.14) | 6 (6.82) | 54 (4.55) | 80 (31.37) |
| Possible only | 295 (19.27) | 20 (22.73) | 125 (10.52) | 150 (58.82) |
| Possible + no recorded causality value[a] | 8 (0.52) | 0 (0.00) | 0 (0.00) | 8 (3.14) |
| Unlikely | 9 (0.59) | 0 (0.00) | 8 (0.67) | 1 (0.39) |
| Conditional/Unclassified | 203 (13.26) | 16 (18.18) | 187 (15.74) | 0 (0.00) |
| Unassessable/Unclassifiable | 62 (4.05) | 0 (0.00) | 62 (5.22) | 0 (0.00) |
| No recorded causality value | 783 (51.14) | 43 (48.86) | 729 (61.36) | 11 (4.31) |
| Multiple completed assessments[b] | 1 (0.07) | 0 (0.00) | 0 (0.00) | 1 (0.39) |
| **B. Effect of the causality restriction at the ICSR level** | | | | |
| Eligible ICSRs before causality restriction | 779 | 23 | 606 | 150 |
| Retained in the causality-restricted analysis, n (%)[c] | 279 (35.82) | 17 (73.91) | 118 (19.47) | 144 (96.00) |
| Excluded by causality restriction, n (%) | 500 (64.18) | 6 (26.09) | 488 (80.53) | 6 (4.00) |
| **C. Reason for complete exclusion, n (% of excluded ICSRs)** | **N = 500** | **N = 6** | **N = 488** | **N = 6** |
| All drug–event relationships had no recorded causality value | 387 (77.40) | 5 (83.33) | 376 (77.05) | 6 (100.00) |
| Recorded causality consisted only of Unlikely, Conditional/Unclassified, Unassessable/Unclassifiable | 111 (22.20) | 0 (0.00) | 111 (22.75) | 0 (0.00) |
| Both missing causality records and causality assessments of Unlikely, Conditional/Unclassified, or Unassessable/Unclassifiable were present | 2 (0.40) | 1 (16.67) | 1 (0.20) | 0 (0.00) |

[a] Eight varenicline drug–event relationships had two source records sharing the same KAERS_NO-DRUG_SEQ-ADR_SEQ combination: one with "Possible" causality assessment and the other with no recorded causality value. These were shown separately to preserve the presence of the missing record.

[b] One varenicline drug–event relationship had two completed causality assessments, Probable/Likely and Possible, for the same drug–event relationship within the same ICSR.

[c] The causality-restricted sensitivity analysis retained drug–event relationships with a recorded WHO-UMC causality assessment of Certain, Probable/Likely, or Possible

**Table S4.** SOC-level disproportionality analysis of adverse event reporting among smoking-cessation medications (n=279 ICSRs)

| Medication | SOC | Target ICSR | Comparator ICSR | PRR (95% CI) | ROR (95% CI) | IC | IC025 | IC975 | $\chi^2$ |
|---|---|---|---|---|---|---|---|---|---|
| Bupropion | Psychiatric disorders | 9/17 (52.94%) | 66/262 (25.19%) | 2.10 (1.28–3.45) | 3.34 (1.24–9.01) | 0.78 | -0.37 | 1.93 | 6.26 |
| | Gastro-intestinal system disorders | 5/17 (29.41%) | 106/262 (40.46%) | 0.73 (0.34–1.54) | 0.61 (0.21–1.79) | -0.44 | -1.79 | 0.92 | 0.81 |
| | Body as a whole - general disorders | 3/17 (17.65%) | 35/262 (13.36%) | 1.32 (0.45–3.86) | 1.39 (0.38–5.08) | 0.2 | -1.43 | 1.83 | 0.25 |
| NRT | Skin and appendages disorders | 33/118 (27.97%) | 7/161 (4.35%) | 6.43 (2.95–14.04) | 8.54 (3.62–20.13) | 0.9 | 0.24 | 1.55 | 30.93 |
| | Central & peripheral nervous system disorders | 18/118 (15.25%) | 26/161 (16.15%) | 0.94 (0.54–1.64) | 0.94 (0.49–1.80) | -0.07 | -0.84 | 0.71 | 0.04 |
| | Psychiatric disorders | 10/118 (8.47%) | 65/161 (40.37%) | 0.21 (0.11–0.39) | 0.14 (0.07–0.28) | -1.58 | -2.5 | -0.66 | 35.25 |
| | Gastro-intestinal system disorders | 32/118 (27.12%) | 79/161 (49.07%) | 0.55 (0.40–0.77) | 0.39 (0.23–0.64) | -0.54 | -1.1 | 0.01 | 13.69 |
| | Metabolic and nutritional disorders | 6/118 (5.08%) | 4/161 (2.48%) | 2.05 (0.59–7.09) | 2.10 (0.58–7.62) | 0.32 | -1.07 | 1.7 | 1.33 |
| | Cardiovascular disorders, general | 3/118 (2.54%) | 4/161 (2.48%) | 1.02 (0.23–4.49) | 1.02 (0.22–4.66) | -0.13 | -1.89 | 1.64 | 0 |
| | Heart rate and rhythm disorders | 5/118 (4.24%) | 2/161 (1.24%) | 3.41 (0.67–17.28) | 3.52 (0.67–18.45) | 0.46 | -1.09 | 2.01 | 2.5 |
| | Respiratory system disorders | 9/118 (7.63%) | 4/161 (2.48%) | 3.07 (0.97–9.73) | 3.24 (0.97–10.79) | 0.54 | -0.65 | 1.73 | 4.05 |
| | Body as a whole - general disorders | 10/118 (8.47%) | 28/161 (17.39%) | 0.49 (0.25–0.96) | 0.44 (0.20–0.95) | -0.66 | -1.64 | 0.31 | 4.6 |
| | Application site disorders | 18/118 (15.25%) | 0/161 (0.00%) | 50.37 (3.07–827.55) | 59.46 (3.54–997.60) | 1.08 | 0.15 | 2.01 | 26.25 |
| | Secondary terms - events | 3/118 (2.54%) | 0/161 (0.00%) | 9.53 (0.50–182.76) | 9.79 (0.50–191.33) | 0.58 | -1.46 | 2.61 | 4.14 |
| Varenicline | Skin and appendages disorders | 6/144 (4.17%) | 34/135 (25.19%) | 0.16 (0.07–0.38) | 0.13 (0.05–0.32) | -1.65 | -2.82 | -0.49 | 25.06 |

| | | | | | | | | |
|---|---|---|---|---|---|---|---|---|
| Central & peripheral nervous system disorders | 24/144 (16.67%) | 20/135 (14.81%) | 1.12 (0.65–1.94) | 1.15 (0.60–2.19) | 0.06 | -0.64 | 0.75 | 0.18 |
| Psychiatric disorders | 56/144 (38.89%) | 19/135 (14.07%) | 2.76 (1.74–4.40) | 3.88 (2.15–7.01) | 0.51 | 0.04 | 0.99 | 21.83 |
| Gastro-intestinal system disorders | 74/144 (51.39%) | 37/135 (27.41%) | 1.88 (1.36–2.58) | 2.80 (1.70–4.62) | 0.36 | -0.03 | 0.75 | 16.73 |
| Metabolic and nutritional disorders | 3/144 (2.08%) | 7/135 (5.19%) | 0.40 (0.11–1.52) | 0.39 (0.10–1.54) | -0.73 | -2.4 | 0.94 | 1.94 |
| Cardiovascular disorders, general | 4/144 (2.78%) | 3/135 (2.22%) | 1.25 (0.28–5.48) | 1.26 (0.28–5.72) | -0.03 | -1.66 | 1.61 | 0.09 |
| Respiratory system disorders | 4/144 (2.78%) | 9/135 (6.67%) | 0.42 (0.13–1.32) | 0.40 (0.12–1.33) | -0.71 | -2.2 | 0.78 | 2.37 |
| Body as a whole - general disorders | 25/144 (17.36%) | 13/135 (9.63%) | 1.80 (0.96–3.38) | 1.97 (0.96–4.04) | 0.31 | -0.4 | 1.02 | 3.54 |

**Table S5.** Disproportionality analysis of PT-Tobacco cessation medication (n=279 ICSRs)

| Medication | Preferred term (PT) | Target, n/N (%) | Comparator, n/N (%) | PRR (95% CI) | ROR (95% CI) | $\chi^2$ | IC | IC025 | IC975 |
|---|---|---|---|---|---|---|---|---|---|
| Bupropion | PRURITUS | 0/17 (0.00) | 21/262 (8.02) | 0.34 (0.02–5.38) | 0.32 (0.02–5.52) | 1.47 | -1.26 | -4.27 | 1.75 |
| | RASH | 0/17 (0.00) | 20/262 (7.63) | 0.36 (0.02–5.66) | 0.34 (0.02–5.83) | 1.4 | -1.22 | -4.24 | 1.79 |
| | URTICARIA | 1/17 (5.88) | 6/262 (2.29) | 2.57 (0.33–20.14) | 2.67 (0.30–23.51) | 0.84 | 0.41 | -1.96 | 2.77 |
| | DIZZINESS | 1/17 (5.88) | 23/262 (8.78) | 0.67 (0.10–4.67) | 0.65 (0.08–5.12) | 0.17 | -0.37 | -2.58 | 1.83 |
| | HEADACHE | 1/17 (5.88) | 14/262 (5.34) | 1.10 (0.15–7.88) | 1.11 (0.14–8.96) | 0.01 | -0.01 | -2.26 | 2.24 |
| | ANOREXIA | 3/17 (17.65) | 6/262 (2.29) | 7.71 (2.11–28.17) | 9.14 (2.07–40.43) | 12.06 | 1.29 | -0.53 | 3.11 |
| | APATHY | 0/17 (0.00) | 4/262 (1.53) | 1.62 (0.09–28.99) | 1.64 (0.08–31.72) | 0.26 | -0.4 | -3.62 | 2.82 |
| | DEPRESSION | 2/17 (11.76) | 3/262 (1.15) | 10.27 (1.84–57.42) | 11.51 (1.79–74.20) | 10.23 | 1.12 | -1.01 | 3.25 |
| | DREAMING ABNORMAL | 0/17 (0.00) | 17/262 (6.49) | 0.42 (0.03–6.66) | 0.40 (0.02–6.95) | 1.17 | -1.1 | -4.13 | 1.93 |
| | INSOMNIA | 5/17 (29.41) | 29/262 (11.07) | 2.66 (1.18–5.99) | 3.35 (1.10–10.18) | 5.02 | 0.9 | -0.52 | 2.31 |
| | SLEEP DISORDER | 0/17 (0.00) | 6/262 (2.29) | 1.12 (0.07–19.17) | 1.13 (0.06–20.84) | 0.4 | -0.53 | -3.68 | 2.61 |
| | SOMNOLENCE | 1/17 (5.88) | 6/262 (2.29) | 2.57 (0.33–20.14) | 2.67 (0.30–23.51) | 0.84 | 0.41 | -1.96 | 2.77 |
| | ABDOMINAL PAIN | 0/17 (0.00) | 6/262 (2.29) | 1.12 (0.07–19.17) | 1.13 (0.06–20.84) | 0.4 | -0.53 | -3.68 | 2.61 |
| | DIARRHOEA | 0/17 (0.00) | 5/262 (1.91) | 1.33 (0.08–23.09) | 1.34 (0.07–25.18) | 0.33 | -0.47 | -3.64 | 2.71 |
| | DYSPEPSIA | 1/17 (5.88) | 23/262 (8.78) | 0.67 (0.10–4.67) | 0.65 (0.08–5.12) | 0.17 | -0.37 | -2.58 | 1.83 |
| | HICCUP | 0/17 (0.00) | 3/262 (1.15) | 2.09 (0.11–38.88) | 2.12 (0.11–42.66) | 0.2 | -0.33 | -3.61 | 2.96 |
| | MOUTH DRY | 1/17 (5.88) | 3/262 (1.15) | 5.14 (0.56–46.80) | 5.40 (0.53–54.84) | 2.54 | 0.6 | -1.89 | 3.09 |
| | NAUSEA | 1/17 (5.88) | 64/262 (24.43) | 0.24 (0.04–1.63) | 0.19 (0.03–1.49) | 3.07 | -1.38 | -3.54 | 0.78 |
| | VOMITING | 0/17 (0.00) | 20/262 (7.63) | 0.36 (0.02–5.66) | 0.34 (0.02–5.83) | 1.4 | -1.22 | -4.24 | 1.79 |
| | HYPERGLYCAEMIA | 0/17 (0.00) | 4/262 (1.53) | 1.62 (0.09–28.99) | 1.64 (0.08–31.72) | 0.26 | -0.4 | -3.62 | 2.82 |
| | HYPERTENSION | 0/17 (0.00) | 5/262 (1.91) | 1.33 (0.08–23.09) | 1.34 (0.07–25.18) | 0.33 | -0.47 | -3.64 | 2.71 |

| | | | | | | | | | |
|---|---|---|---|---|---|---|---|---|---|
| | PALPITATION | 0/17 (0.00) | 5/262 (1.91) | 1.33 (0.08–23.09) | 1.34 (0.07–25.18) | 0.33 | -0.47 | -3.64 | 2.71 |
| | DYSPNOEA | 0/17 (0.00) | 3/262 (1.15) | 2.09 (0.11–38.88) | 2.12 (0.11–42.66) | 0.2 | -0.33 | -3.61 | 2.96 |
| | PHARYNGITIS | 0/17 (0.00) | 3/262 (1.15) | 2.09 (0.11–38.88) | 2.12 (0.11–42.66) | 0.2 | -0.33 | -3.61 | 2.96 |
| | CHEST PAIN | 0/17 (0.00) | 8/262 (3.05) | 0.86 (0.05–14.30) | 0.86 (0.05–15.44) | 0.53 | -0.65 | -3.76 | 2.45 |
| | MALAISE | 2/17 (11.76) | 17/262 (6.49) | 1.81 (0.46–7.21) | 1.92 (0.41–9.10) | 0.7 | 0.4 | -1.49 | 2.29 |
| | APPLICATION SITE REACTION | 0/17 (0.00) | 17/262 (6.49) | 0.42 (0.03–6.66) | 0.40 (0.02–6.95) | 1.17 | -1.1 | -4.13 | 1.93 |
| | DRUG QUALITY PROBLEM | 0/17 (0.00) | 3/262 (1.15) | 2.09 (0.11–38.88) | 2.12 (0.11–42.66) | 0.2 | -0.33 | -3.61 | 2.96 |
| NRT | PRURITUS | 19/118 (16.10) | 2/161 (1.24) | 12.96 (3.08–54.57) | 15.26 (3.48–66.93) | 21.6 | 0.96 | 0.08 | 1.85 |
| | RASH | 19/118 (16.10) | 1/161 (0.62) | 25.92 (3.52–190.95) | 30.71 (4.05–232.98) | 24.52 | 1.02 | 0.13 | 1.92 |
| | URTICARIA | 5/118 (4.24) | 2/161 (1.24) | 3.41 (0.67–17.28) | 3.52 (0.67–18.45) | 2.5 | 0.46 | -1.09 | 2.01 |
| | DIZZINESS | 11/118 (9.32) | 13/161 (8.07) | 1.15 (0.54–2.49) | 1.17 (0.50–2.71) | 0.13 | 0.06 | -0.94 | 1.06 |
| | HEADACHE | 5/118 (4.24) | 10/161 (6.21) | 0.68 (0.24–1.94) | 0.67 (0.22–2.01) | 0.52 | -0.37 | -1.74 | 1.01 |
| | ANOREXIA | 0/118 (0.00) | 9/161 (5.59) | 0.07 (0.00–1.22) | 0.07 (0.00–1.18) | 6.82 | -2.38 | -5.4 | 0.64 |
| | APATHY | 0/118 (0.00) | 4/161 (2.48) | 0.15 (0.01–2.78) | 0.15 (0.01–2.77) | 2.97 | -1.63 | -4.79 | 1.52 |
| | DEPRESSION | 0/118 (0.00) | 5/161 (3.11) | 0.12 (0.01–2.22) | 0.12 (0.01–2.19) | 3.73 | -1.82 | -4.93 | 1.29 |
| | DREAMING ABNORMAL | 0/118 (0.00) | 17/161 (10.56) | 0.04 (0.00–0.64) | 0.03 (0.00–0.59) | 13.27 | -3.1 | -6.06 | -0.14 |
| | INSOMNIA | 5/118 (4.24) | 29/161 (18.01) | 0.24 (0.09–0.59) | 0.20 (0.08–0.54) | 12.07 | -1.39 | -2.66 | -0.12 |
| | SLEEP DISORDER | 0/118 (0.00) | 6/161 (3.73) | 0.10 (0.01–1.84) | 0.10 (0.01–1.81) | 4.49 | -1.98 | -5.06 | 1.1 |

| | | | | | | | | |
|---|---|---|---|---|---|---|---|---|
| SOMNOLENCE | 2/118 (1.69) | 5/161 (3.11) | 0.55 (0.11–2.76) | 0.54 (0.10–2.82) | 0.55 | -0.54 | -2.49 | 1.41 |
| ABDOMINAL PAIN | 3/118 (2.54) | 3/161 (1.86) | 1.36 (0.28–6.64) | 1.37 (0.27–6.93) | 0.15 | 0.02 | -1.78 | 1.82 |
| DIARRHOEA | 2/118 (1.69) | 3/161 (1.86) | 0.91 (0.15–5.36) | 0.91 (0.15–5.52) | 0.01 | -0.23 | -2.27 | 1.8 |
| DYSPEPSIA | 5/118 (4.24) | 19/161 (11.80) | 0.36 (0.14–0.93) | 0.33 (0.12–0.91) | 4.96 | -0.94 | -2.24 | 0.36 |
| HICCUP | 3/118 (2.54) | 0/161 (0.00) | 9.53 (0.50–182.76) | 9.79 (0.50–191.33) | 4.14 | 0.58 | -1.46 | 2.61 |
| MOUTH DRY | 0/118 (0.00) | 4/161 (2.48) | 0.15 (0.01–2.78) | 0.15 (0.01–2.77) | 2.97 | -1.63 | -4.79 | 1.52 |
| NAUSEA | 18/118 (15.25) | 47/161 (29.19) | 0.52 (0.32–0.85) | 0.44 (0.24–0.80) | 7.4 | -0.6 | -1.34 | 0.14 |
| VOMITING | 8/118 (6.78) | 12/161 (7.45) | 0.91 (0.38–2.15) | 0.90 (0.36–2.28) | 0.05 | -0.13 | -1.27 | 1.01 |
| HYPERGLYCAEMIA | 4/118 (3.39) | 0/161 (0.00) | 12.25 (0.67–225.40) | 12.69 (0.68–238.11) | 5.54 | 0.69 | -1.13 | 2.51 |
| HYPERTENSION | 3/118 (2.54) | 2/161 (1.24) | 2.05 (0.35–12.06) | 2.07 (0.34–12.61) | 0.65 | 0.18 | -1.67 | 2.04 |
| PALPITATION | 4/118 (3.39) | 1/161 (0.62) | 5.46 (0.62–48.20) | 5.61 (0.62–50.89) | 2.97 | 0.51 | -1.24 | 2.25 |
| DYSPNOEA | 3/118 (2.54) | 0/161 (0.00) | 9.53 (0.50–182.76) | 9.79 (0.50–191.33) | 4.14 | 0.58 | -1.46 | 2.61 |
| PHARYNGITIS | 3/118 (2.54) | 0/161 (0.00) | 9.53 (0.50–182.76) | 9.79 (0.50–191.33) | 4.14 | 0.58 | -1.46 | 2.61 |
| CHEST PAIN | 4/118 (3.39) | 4/161 (2.48) | 1.36 (0.35–5.34) | 1.38 (0.34–5.62) | 0.2 | 0.06 | -1.54 | 1.67 |
| MALAISE | 0/118 (0.00) | 19/161 (11.80) | 0.03 (0.00–0.57) | 0.03 (0.00–0.52) | 14.94 | -3.23 | -6.18 | -0.28 |
| APPLICATION SITE REACTION | 17/118 (14.41) | 0/161 (0.00) | 47.65 (2.89–784.48) | 55.69 (3.31–936.28) | 24.7 | 1.07 | 0.12 | 2.02 |
| DRUG QUALITY | 3/118 | 0/161 (0.00) | 9.53 (0.50–182.76) | 9.79 (0.50– | 4.14 | 0.58 | -1.46 | 2.61 |

| | | | | | | | | | |
|---|---|---|---|---|---|---|---|---|---|
| | PROBLEM | (2.54) | | | 191.33) | | | | |
| Varenicline | PRURITUS | 2/144 (1.39) | 19/135 (14.07) | 0.10 (0.02–0.42) | 0.09 (0.02–0.38) | 16.11 | -2.03 | -3.8 | -0.27 |
| | RASH | 1/144 (0.69) | 19/135 (14.07) | 0.05 (0.01–0.36) | 0.04 (0.01–0.32) | 18.74 | -2.56 | -4.68 | -0.43 |
| | URTICARIA | 1/144 (0.69) | 6/135 (4.44) | 0.16 (0.02–1.28) | 0.15 (0.02–1.27) | 4.01 | -1.35 | -3.62 | 0.92 |
| | DIZZINESS | 12/144 (8.33) | 12/135 (8.89) | 0.94 (0.44–2.01) | 0.93 (0.40–2.15) | 0.03 | -0.09 | -1.06 | 0.88 |
| | HEADACHE | 9/144 (6.25) | 6/135 (4.44) | 1.41 (0.51–3.85) | 1.43 (0.50–4.14) | 0.45 | 0.12 | -1.03 | 1.27 |
| | ANOREXIA | 6/144 (4.17) | 3/135 (2.22) | 1.87 (0.48–7.35) | 1.91 (0.47–7.81) | 0.84 | 0.19 | -1.22 | 1.6 |
| | APATHY | 4/144 (2.78) | 0/135 (0.00) | 8.44 (0.46–155.33) | 8.68 (0.46–162.76) | 3.8 | 0.49 | -1.33 | 2.31 |
| | DEPRESSION | 3/144 (2.08) | 2/135 (1.48) | 1.41 (0.24–8.29) | 1.41 (0.23–8.60) | 0.14 | -0.03 | -1.88 | 1.83 |
| | DREAMING ABNORMAL | 17/144 (11.81) | 0/135 (0.00) | 32.83 (1.99–540.61) | 37.20 (2.21–625.01) | 16.97 | 0.82 | -0.13 | 1.76 |
| | INSOMNIA | 24/144 (16.67) | 10/135 (7.41) | 2.25 (1.12–4.53) | 2.50 (1.15–5.45) | 5.58 | 0.4 | -0.33 | 1.14 |
| | SLEEP DISORDER | 6/144 (4.17) | 0/135 (0.00) | 12.19 (0.69–214.40) | 12.72 (0.71–227.98) | 5.75 | 0.61 | -0.92 | 2.14 |
| | SOMNOLENCE | 4/144 (2.78) | 3/135 (2.22) | 1.25 (0.28–5.48) | 1.26 (0.28–5.72) | 0.09 | -0.03 | -1.66 | 1.61 |
| | ABDOMINAL PAIN | 3/144 (2.08) | 3/135 (2.22) | 0.94 (0.19–4.57) | 0.94 (0.19–4.72) | 0.01 | -0.2 | -2 | 1.6 |
| | DIARRHOEA | 3/144 (2.08) | 2/135 (1.48) | 1.41 (0.24–8.29) | 1.41 (0.23–8.60) | 0.14 | -0.03 | -1.88 | 1.83 |
| | DYSPEPSIA | 18/144 (12.50) | 6/135 (4.44) | 2.81 (1.15–6.87) | 3.07 (1.18–7.99) | 5.75 | 0.46 | -0.4 | 1.32 |
| | HICCUP | 0/144 (0.00) | 3/135 (2.22) | 0.13 (0.01–2.57) | 0.13 (0.01–2.56) | 3.23 | -1.61 | -4.83 | 1.61 |

| | | | | | | | | |
|---|---|---|---|---|---|---|---|---|
| MOUTH DRY | 3/144 (2.08) | 1/135 (0.74) | 2.81 (0.30–26.71) | 2.85 (0.29–27.75) | 0.89 | 0.17 | -1.76 | 2.09 |
| NAUSEA | 46/144 (31.94) | 19/135 (14.07) | 2.27 (1.40–3.67) | 2.87 (1.58–5.21) | 12.45 | 0.43 | -0.09 | 0.95 |
| VOMITING | 12/144 (8.33) | 8/135 (5.93) | 1.41 (0.59–3.33) | 1.44 (0.57–3.65) | 0.61 | 0.14 | -0.86 | 1.15 |
| HYPERGLYCAEMIA | 0/144 (0.00) | 4/135 (2.96) | 0.10 (0.01–1.92) | 0.10 (0.01–1.90) | 4.33 | -1.83 | -4.99 | 1.32 |
| HYPERTENSION | 2/144 (1.39) | 3/135 (2.22) | 0.62 (0.11–3.68) | 0.62 (0.10–3.77) | 0.27 | -0.44 | -2.47 | 1.59 |
| PALPITATION | 1/144 (0.69) | 4/135 (2.96) | 0.23 (0.03–2.07) | 0.23 (0.03–2.08) | 2.04 | -1.03 | -3.37 | 1.32 |
| DYSPNOEA | 0/144 (0.00) | 3/135 (2.22) | 0.13 (0.01–2.57) | 0.13 (0.01–2.56) | 3.23 | -1.61 | -4.83 | 1.61 |
| PHARYNGITIS | 0/144 (0.00) | 3/135 (2.22) | 0.13 (0.01–2.57) | 0.13 (0.01–2.56) | 3.23 | -1.61 | -4.83 | 1.61 |
| CHEST PAIN | 4/144 (2.78) | 4/135 (2.96) | 0.94 (0.24–3.67) | 0.94 (0.23–3.82) | 0.01 | -0.17 | -1.77 | 1.43 |
| MALAISE | 17/144 (11.81) | 2/135 (1.48) | 7.97 (1.88–33.84) | 8.90 (2.02–39.31) | 11.7 | 0.68 | -0.24 | 1.6 |
| APPLICATION SITE REACTION | 0/144 (0.00) | 17/135 (12.59) | 0.03 (0.00–0.44) | 0.02 (0.00–0.39) | 19.31 | -3.35 | -6.31 | -0.4 |
| DRUG QUALITY PROBLEM | 0/144 (0.00) | 3/135 (2.22) | 0.13 (0.01–2.57) | 0.13 (0.01–2.56) | 3.23 | -1.61 | -4.83 | 1.61 |

**Table S6.** Sensitivity analysis of signals of disproportionate reporting using an alternative Gamma–Poisson shrinkage approach

| Medication | SOC | Target ICSR | Comparator ICSR | PRR (95% CI) | ROR (95% CI) | Gamma–Poisson estimate | Lower 95% CrI | Upper 95% CrI | χ² |
|---|---|---|---|---|---|---|---|---|---|
| Bupropion | Skin and appendages disorders | 5/23 (21.74%) | 142/756 (18.78%) | 1.16 (0.53–2.55) | 1.20 (0.44–3.29) | 0.18 | -1.34 | 1.18 | 0.13 |
| | Central & peripheral nervous system disorders | 5/23 (21.74%) | 128/756 (16.93%) | 1.28 (0.58–2.83) | 1.36 (0.50–3.74) | 0.31 | -1.21 | 1.31 | 0.36 |
| | Psychiatric disorders | 13/23 (56.52%) | 125/756 (16.53%) | 3.42 (2.31–5.06) | 6.56 (2.81–15.30) | 1.56 | 0.67 | 2.24 | 24.48 |
| | Gastro-intestinal system disorders | 10/23 (43.48%) | 206/756 (27.25%) | 1.60 (0.99–2.58) | 2.05 (0.89–4.76) | 0.61 | -0.42 | 1.37 | 2.93 |
| | Respiratory system disorders | 3/23 (13.04%) | 46/756 (6.08%) | 2.14 (0.72–6.39) | 2.32 (0.66–8.08) | 0.85 | -1.2 | 2.04 | 1.83 |
| | Body as a whole - general disorders | 5/23 (21.74%) | 122/756 (16.14%) | 1.35 (0.61–2.97) | 1.44 (0.53–3.96) | 0.37 | -1.16 | 1.37 | 0.51 |
| NRT | Skin and appendages disorders | 135/606 (22.28%) | 12/173 (6.94%) | 3.21 (1.82–5.65) | 3.85 (2.08–7.13) | 0.24 | -0.02 | 0.47 | 20.69 |
| | Musculo-skeletal system disorders | 19/606 (3.14%) | 3/173 (1.73%) | 1.81 (0.54–6.04) | 1.83 (0.54–6.27) | 0.15 | -0.57 | 0.72 | 0.96 |
| | Central & peripheral nervous system disorders | 102/606 (16.83%) | 31/173 (17.92%) | 0.94 (0.65–1.35) | 0.93 (0.60–1.44) | -0.02 | -0.31 | 0.25 | 0.11 |
| | Vision disorders | 11/606 (1.82%) | 0/173 (0.00%) | 6.59 (0.39–111.33) | 6.70 (0.39–114.30) | 0.34 | -0.63 | 1.07 | 3.19 |
| | Hearing and vestibular disorders | 7/606 (1.16%) | 2/173 (1.16%) | 1.00 (0.21–4.77) | 1.00 (0.21–4.85) | 0 | -1.26 | 0.87 | 0 |
| | Special senses other, disorders | 6/606 (0.99%) | 0/173 (0.00%) | 3.73 (0.21–65.83) | 3.76 (0.21–67.01) | 0.33 | -1.05 | 1.26 | 1.73 |
| | Psychiatric disorders | 69/606 (11.39%) | 69/173 (39.88%) | 0.29 (0.21–0.38) | 0.19 (0.13–0.29) | -0.63 | -0.99 | -0.31 | 74.98 |

| | | | | | | | | |
|---|---|---|---|---|---|---|---|---|
| Gastro-intestinal system disorders | 130/606 (21.45%) | 86/173 (49.71%) | 0.43 (0.35–0.53) | 0.28 (0.19–0.39) | -0.37 | -0.63 | -0.13 | 53.63 |
| Liver and biliary system disorders | 3/606 (0.50%) | 0/173 (0.00%) | 2.01 (0.10–38.66) | 2.01 (0.10–39.15) | 0.3 | -1.75 | 1.5 | 0.86 |
| Metabolic and nutritional disorders | 22/606 (3.63%) | 4/173 (2.31%) | 1.57 (0.55–4.50) | 1.59 (0.54–4.68) | 0.12 | -0.55 | 0.66 | 0.72 |
| Cardiovascular disorders, general | 14/606 (2.31%) | 5/173 (2.89%) | 0.80 (0.29–2.19) | 0.79 (0.28–2.24) | -0.08 | -0.93 | 0.58 | 0.19 |
| Heart rate and rhythm disorders | 19/606 (3.14%) | 2/173 (1.16%) | 2.71 (0.64–11.53) | 2.77 (0.64–12.00) | 0.21 | -0.51 | 0.79 | 2.01 |
| Vascular (extracardiac) disorders | 11/606 (1.82%) | 0/173 (0.00%) | 6.59 (0.39–111.33) | 6.70 (0.39–114.30) | 0.34 | -0.63 | 1.07 | 3.19 |
| Respiratory system disorders | 41/606 (6.77%) | 8/173 (4.62%) | 1.46 (0.70–3.06) | 1.50 (0.69–3.26) | 0.1 | -0.37 | 0.51 | 1.05 |
| Red blood cell disorders | 3/606 (0.50%) | 0/173 (0.00%) | 2.01 (0.10–38.66) | 2.01 (0.10–39.15) | 0.3 | -1.75 | 1.5 | 0.86 |
| White cell and RES* disorders | 6/606 (0.99%) | 0/173 (0.00%) | 3.73 (0.21–65.83) | 3.76 (0.21–67.01) | 0.33 | -1.05 | 1.26 | 1.73 |
| Platelet, bleeding & clotting disorders | 4/606 (0.66%) | 0/173 (0.00%) | 2.58 (0.14–47.69) | 2.59 (0.14–48.38) | 0.32 | -1.42 | 1.4 | 1.15 |
| Urinary system disorders | 4/606 (0.66%) | 4/173 (2.31%) | 0.29 (0.07–1.13) | 0.28 (0.07–1.13) | -0.58 | -2.32 | 0.5 | 3.61 |
| Reproductive disorders, female | 3/606 (0.50%) | 0/173 (0.00%) | 2.01 (0.10–38.66) | 2.01 (0.10–39.15) | 0.3 | -1.75 | 1.5 | 0.86 |
| Body as a whole - general disorders | 96/606 (15.84%) | 31/173 (17.92%) | 0.88 (0.61–1.28) | 0.86 (0.55–1.35) | -0.04 | -0.34 | 0.23 | 0.43 |
| Application site disorders | 111/606 (18.32%) | 0/173 (0.00%) | 63.92 (3.99–1023.16) | 78.08 (4.83–1262.97) | 0.36 | 0.08 | 0.62 | 36.95 |
| Secondary terms - events | 67/606 (11.06%) | 1/173 (0.58%) | 19.13 (2.67–136.79) | 21.38 (2.95–155.17) | 0.34 | -0.03 | 0.66 | 18.55 |

| | | | | | | | | | |
|---|---|---|---|---|---|---|---|---|---|
| Varenicline | Skin and appendages disorders | 7/150 (4.67%) | 140/629 (22.26%) | 0.21 (0.10–0.44) | 0.17 (0.08–0.37) | -1.94 | -3.2 | -1.07 | 24.48 |
| | Central & peripheral nervous system disorders | 26/150 (17.33%) | 107/629 (17.01%) | 1.02 (0.69–1.50) | 1.02 (0.64–1.64) | 0.02 | -0.59 | 0.52 | 0.01 |
| | Psychiatric disorders | 56/150 (37.33%) | 82/629 (13.04%) | 2.86 (2.14–3.82) | 3.97 (2.65–5.95) | 1.06 | 0.66 | 1.41 | 49.05 |
| | Gastro-intestinal system disorders | 76/150 (50.67%) | 140/629 (22.26%) | 2.28 (1.84–2.82) | 3.59 (2.47–5.20) | 0.86 | 0.52 | 1.17 | 48.78 |
| | Metabolic and nutritional disorders | 3/150 (2.00%) | 23/629 (3.66%) | 0.55 (0.17–1.80) | 0.54 (0.16–1.82) | -0.65 | -2.7 | 0.54 | 1.03 |
| | Cardiovascular disorders, general | 5/150 (3.33%) | 14/629 (2.23%) | 1.50 (0.55–4.09) | 1.51 (0.54–4.27) | 0.4 | -1.12 | 1.4 | 0.62 |
| | Respiratory system disorders | 5/150 (3.33%) | 44/629 (7.00%) | 0.48 (0.19–1.18) | 0.46 (0.18–1.18) | -0.85 | -2.38 | 0.14 | 2.76 |
| | Urinary system disorders | 4/150 (2.67%) | 4/629 (0.64%) | 4.19 (1.06–16.58) | 4.28 (1.06–17.32) | 1.14 | -0.6 | 2.22 | 4.91 |
| | Body as a whole - general disorders | 26/150 (17.33%) | 101/629 (16.06%) | 1.08 (0.73–1.60) | 1.10 (0.68–1.76) | 0.09 | -0.52 | 0.59 | 0.14 |
